# Synthesis and characterization of cyclobutenedione–bithiophene π-conjugated polymers: acetal-protecting strategy for Kumada–Tamao–Corriu coupling polymerization between aryl bromide and Grignard reagents†

**DOI:** 10.1039/c9ra08275a

**Published:** 2019-12-11

**Authors:** Tomoyuki Ohishi, Takuma Sone, Kohei Oda, Akihiro Yokoyama

**Affiliations:** Department of Materials and Life Science, Faculty of Science and Technology, Seikei University 3-3-1 Kichijoji-Kitamachi Musashino Tokyo 180-8633 Japan t-ohishi@st.seikei.ac.jp ayokoyama@st.seikei.ac.jp

## Abstract

Cyclobutenedione is an aromatic ring that exhibits strong electron-withdrawing properties but is susceptible to undesired reactions with nucleophiles. Herein, Kumada–Tamao–Corriu coupling polymerization of a cyclobutenedione monomer whose carbonyl groups are protected as acetals was achieved. Hydrolysis of the acetals afforded donor–acceptor type π-conjugated polymers consisting of cyclobutenedione as an acceptor unit and bithiophene as a donor unit. The acetal-protected monomer was also subjected to Suzuki–Miyaura coupling polymerization. The absorption and emission spectra of the deprotected polymers shifted to the longer wavelength compared with the acetal-protected polymers.

## Abstract 2

Donor–acceptor-type π-conjugated polymers consisting of cyclobutenedione as an acceptor and bithiophene as a donor were synthesized by Kumada–Tamao–Corriu coupling polymerization of an acetal-protected monomer.

## Graphical abstract

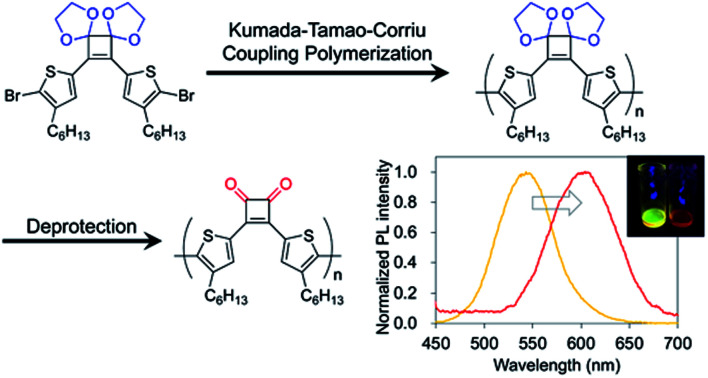

## Introduction

Cyclobutenedione is a four-membered aromatic ring with two carbonyl groups that render this unit strongly electron-withdrawing. Among the various cyclobutenedione derivatives, the most well-known is strongly acidic 3,4-dihydroxy-3-cyclobutene-1,2-dione (squaric acid).^1^ Recently, squaric acid bisamides (squaramides) have attracted significant attention and have been used as chemosensors^2^ and organocatalysts.^3^ The cyclobutenedione unit has been used for pyrolytic and photolytic syntheses of functionalized benzoquinone and phenol^4^ as well as cyclo[*n*]carbon,^5^ and can serve as a fixed *cis*-vinylene linker in photochromic derivatives^6^ and anticancer agents.^7^

In the field of polymer chemistry, the synthesis of polysquaramide is commonly achieved by polycondensation of squaric acids or squaric acid diesters and diamines.^8^ The reactions between squaric acid diesters and amines proceed under mild conditions.^1,2*d*^ Furthermore, the synthesis of π-conjugated cyclobutenedione polymers has been reported by Suh's group, who synthesized cyclobutenedione-containing poly(phenylenevinylene)s by dehalogenation polycondensation and Heck coupling polymerization.^9^ These polymers showed blue photoluminescence in solution. Huang *et al.* reported the synthesis of π-conjugated cyclobutenedione polymers *via* Suzuki–Miyaura and Stille coupling polymerization.^10^ The prepared polymers exhibited broad and strong absorption bands in UV-vis region and high electron affinity.

Inspired by these studies, we expected that the cyclobutenedione unit could be used as an electron acceptor in the same manner as maleimide^11^ and donor–acceptor-type π-conjugated cyclobutenedione polymers would exhibit interesting properties. However, only a few reports regarding the synthesis of π-conjugated polymers with cyclobutenedione in the main chain have been published to date.^9,10^ This is likely due to the unstable and reactive nature of cyclobutenedione, which can be transformed into highly reactive bisketenes under heating and light irradiation.^12^ These compounds can subsequently be subjected to Diels–Alder cycloaddition, dimerization, and coupling with alcohols.^13^ The cyclobutenedione units in polymer main chains can also be converted into bisketenes.^14^ In addition, nucleophiles including organolithium and Grignard reagents react with the carbonyl groups of cyclobutenedione.^15^ If a polymerization method applicable to the synthesis of π-conjugated cyclobutenedione polymers can be developed, it can provide a facile method with which to achieve various donor–acceptor cyclobutenedione polymers.

Herein, we describe two approaches to donor–acceptor-type π-conjugated polymers consisting of a cyclobutenedione acceptor and bithiophene donor. The first approach involved direct coupling of the cyclobutenedione unit and aromatic rings. Because the reactivity of the cyclobutenedione carbon atoms at the 3- and 4-positions is similar to carbonyl carbons, the desired cyclobutenedione polymer could likely be synthesized using similar methods to the synthesis of aryl ketones. Therefore, Suzuki–Miyaura coupling of 3,4-dichloro-3-cyclobutene-1,2-dione (squaric acid dichloride) (1a) or Liebeskind–Srogl coupling of 3,4-bis[(4-methoxyphenyl)thio]-3-cyclobutene-1,2-dione (squaric acid thioester) (1b) under ketone synthesis conditions were studied as model reactions. The second method used acetal-protected cyclobutenedione monomer, which was subsequently subjected to Kumada–Tamao–Corriu and Suzuki–Miyaura coupling polymerizations. We choose acetal as a protecting group, because it is stable under basic conditions but easily hydrolyzed under aqueous acidic conditions. Finally, the optical properties of the obtained cyclobutenedione polymers were investigated.

## Experimental

### Measurements

The ^1^H and ^13^C NMR spectra were obtained using a JEOL ECA-500 instrument. The internal standards used for the ^1^H and ^13^C NMR spectra in CDCl_3_ were tetramethylsilane (0.00 ppm) and the midpoint of CDCl_3_ (77.0 ppm), respectively. The *M*_n_ and *M*_w_/*M*_n_ values of the polymers were measured using a TOSOH HLC-8220 gel permeation chromatography (GPC) unit (eluent, THF; calibration, polystyrene standards) with two TSK-gel columns (Multipore H_XL_-M) and a TOSOH HLC-8320 GPC unit (eluent, CHCl_3_; calibration, polystyrene standards) containing two TSK-gel columns (2 × SuperMultiporeHZ-M). IR spectra were recorded using a JASCO FT/IR-470 plus and UV-vis spectra were recorded using a JASCO V-650. The fluorescence spectra were recorded using a JASCO FP-6500 instrument. Electrospray ionization (ESI) mass spectra were recorded on a Thermo Fisher Scientific Q Exactive Hybrid Quadrupole-Orbitrap Mass Spectrometer.

### Materials

3,4-Dihydroxy-3-cyclobutene-1,2-dione (TCI), 4,4,5,5-tetramethyl-1,3,2-dioxaborolane (TCI), tri(2-furyl)phosphine (TFP; TCI), *p*-toluenesulfonic acid monohydrate (TsOH·H_2_O; TCI), cesium carbonate (Cs_2_CO_3_; TCI), 9,9-dioctyl-9*H*-fluorene-2,7-diboronic acid bis(pinacol) ester (TCI), phenylboronic acid (Kanto), 2-thiopheneboronic acid (Kanto), triphenylphosphine (PPh_3_; Kanto), 2-dicyclohexylphosphino-2′,6′-dimethoxybiphenyl (SPhos; Kanto), tri-potassium phosphate *n*-hydrate (K_3_PO_4_·*n*H_2_O; Kanto), lithium chloride (LiCl; Kanto), 4-methoxythiophenol (Aldrich), 2.0 M solution of isopropylmagnesium chloride (^i^PrMgCl) in THF (Aldrich), 1,4-benzenediboronic acid bis(pinacol) ester (Wako), sodium carbonate (Na_2_CO_3_; Wako), trifluoroacetic acid (TFA; Wako), dehydrated DMF (Wako), dehydrated benzene (Kanto), dehydrated toluene (Wako), dehydrated THF (Wako), dehydrated diethyl ether (Wako), copper(i)-2-thiophenecarboxylate (CuTC; TCI), [1,3-bis(diphenylphosphino)propane]nickel(ii) dichloride (Ni(dppp)Cl_2_; TCI), tetrakis(triphenylphosphine)palladium(0) (Pd(PPh_3_)_4_; Kanto), bis(triphenylphosphine)palladium(ii) dichloride (PdCl_2_(PPh_3_)_2_; Aldrich), bis(dibenzylideneacetone)palladium(0) (Pd(dba)_2_; Aldrich), tris(dibenzylideneacetone)dipalladium(0) (Pd_2_(dba)_3_; Aldrich), and palladium(ii) acetate (Pd(OAc)_2_; Wako) were used as-received without purification. Squaric acid dichloride (1a)^16^ and 3,4-bis[(4-methoxyphenyl)thio]-3-cyclobutene-1,2-dione (1b)^17^ were synthesized according to procedures described in the literature.

### Typical procedure of Suzuki–Miyaura coupling between squaric acid dichloride (1a) and phenylboronic acid (2a)

A round-bottomed flask equipped with a three-way stopcock was heated under reduced pressure and subsequently cooled to room temperature under an argon atmosphere. Then, 2a (146 mg, 1.20 mmol), PdCl_2_(PPh_3_)_2_ (7 mg, 0.01 mmol), K_3_PO_4_·*n*H_2_O (718 mg, 3.00 mmol), and 1a (76 mg, 0.50 mmol) were added to the flask, and the atmosphere in the flask was replaced with argon. After addition of dry toluene (2.5 mL) to the flask using a syringe, the flask was evacuated and filled with argon three times, and the reaction mixture was stirred at 110 °C for 3 h. Subsequently, ethyl acetate was added at room temperature and the mixture was washed successively with saturated aqueous NaHCO_3_, water, and brine, and dried over anhydrous MgSO_4_. The mixture was filtered using Celite and the solvent was distilled off under reduced pressure. The crude product (brown solid, 70 mg) was analyzed by ^1^H NMR.

### Typical procedure of coupling reaction between 1a and 2a in the presence of copper(i)-2-thiophenecarboxylate (CuTC)

A round-bottomed flask equipped with a three-way stopcock was heated under reduced pressure and subsequently cooled to room temperature under an argon atmosphere. Then, 2a (244 mg, 2.00 mmol), PPh_3_ (14 mg, 0.053 mmol), 1a (75 mg, 0.50 mmol), and CuTC (95 mg, 0.50 mmol) were added to the flask and the atmosphere was replaced with argon. Pd(dba)_2_ (14 mg, 0.024 mmol) was added to the flask and the atmosphere in the flask was replaced with argon. After addition of dry diethyl ether (15 mL) to the flask using a syringe, the flask was evacuated and filled with argon three times, and the mixture was subsequently stirred at room temperature for 49 h and filtered using Celite at room temperature. The solvent was distilled off under reduced pressure and the crude product (brown solid, 393 mg) was analyzed by ^1^H NMR.

### Typical procedure of Liebeskind–Srogl coupling between 3,4-bis[(4-methoxyphenyl)thio]-3-cyclobutene-1,2-dione (1b) and 2-thiopheneboronic acid (2b)

A round-bottomed flask equipped with a three-way stopcock was heated under reduced pressure and subsequently cooled to room temperature under an argon atmosphere. Then, 1b (70 mg, 0.20 mmol) and dry THF (10 mL) were added to the flask and the solution was deoxygenated by bubbling argon for 5 min. CuTC (95 mg, 0.50 mmol) and TFP (4 mg, 0.02 mmol) were added to the flask, which was then evacuated and filled with argon three times. Pd_2_(dba)_3_ (7 mg, 0.007 mmol) and 2-thiopheneboronic acid (2b) (51 mg, 0.40 mmol) were added to the flask, which was then evacuated and filled with argon three times. The mixture was stirred at 55 °C for 19 h, cooled to room temperature, and filtered using Celite and SiO_2_. The filtrate solvent was distilled off under reduced pressure. The crude material was purified *via* column chromatography on a silica gel (ethyl acetate/hexane = 1/5) and recrystallization from ethyl acetate to afford 3b (45 mg, 56%) as an ocher solid: mp 202.0–202.7 °C. ^1^H NMR (500 MHz, CDCl_3_) *δ* 8.43 (dd, *J* = 4.0 and 1.1 Hz, 2H, thiophene-H), 7.97 (dd, *J* = 4.9 and 1.0 Hz, 2H, thiophene-H), 7.39 (dd, *J* = 4.9 and 3.7 Hz, 2H, thiophene-H); ^13^C NMR (126 MHz, CDCl_3_) *δ* 193.0, 173.1, 135.2, 134.0, 129.3; IR (KBr) 1765, 1754, 1568, 1513, 1418, 1405, 1387, 1372, 1154, 1116, 1062, 868, 726 cm^−1^; ESI-MS calcd for C_12_H_6_NaO_2_S_2_^+^*m*/*z* 268.9701 (M + Na)^+^, found *m*/*z* 268.9691.

### Polymerization of monomer 4 by Kumada–Tamao–Corriu coupling

A round-bottomed flask equipped with a three-way stopcock containing LiCl (33 mg, 0.78 mmol) was heated under reduced pressure and subsequently cooled to room temperature under a nitrogen atmosphere. A solution of 4 (396 mg, 0.600 mmol) in dry THF (3.3 mL) was added to the flask under a nitrogen atmosphere and the mixture was cooled to −20 °C and stirred for 20 min. A 2.0 M solution of ^i^PrMgCl in THF (0.30 mL, 0.60 mmol) was added and the mixture was stirred at −20 °C for 1 h. After a suspension of Ni(dppp)Cl_2_ (13 mg, 0.024 mmol) in THF (1.2 mL) was added to the flask using a syringe, the mixture was stirred at 0 °C for 2 h and 40 °C for 48 h afterwards. The reaction was quenched by adding methanol and the solvent was subsequently removed under vacuum. CH_2_Cl_2_ was added to the residue and the insoluble material was removed by suction filtration and thoroughly washed with CH_2_Cl_2_. After the removal of the solvent *in vacuo* from the filtrate, the residue was again dissolved in CH_2_Cl_2_ and poured into methanol with vigorous stirring. The precipitated polymer was collected and dried *in vacuo* to afford P1 (229 mg, 76%). *M*_n_ = 7400, *M*_w_/*M*_n_ = 10.8. ^1^H NMR (500 MHz, CDCl_3_) *δ* 7.42 (br s, 2H), 4.26 (m, 4H), 4.06 (m, 4H), 2.53 (t, *J* = 7.2 Hz, 4H), 1.57–1.51 (m, 4H), 1.27–1.21 (m, 12H), 0.86–0.79 (m, 6H); ^13^C NMR (126 MHz, CDCl_3_) *δ* 143.2, 133.0, 131.8, 130.8, 130.4, 114.1, 65.9, 31.6, 30.7, 29.1, 28.8, 22.6, 14.0.

### Polymerization of monomer 4 and 5 by Suzuki–Miyaura coupling

A round-bottomed flask equipped with a three-way stopcock was heated under reduced pressure and subsequently cooled to room temperature under an argon atmosphere. Then, 4 (132 mg, 0.200 mmol), 1,4-benzenediboronic acid bis(pinacol) ester (5a) (66 mg, 0.20 mmol), and Cs_2_CO_3_ (286 mg, 0.880 mmol) were added to the flask, which was evacuated and filled with argon three times. Pd(PPh_3_)_4_ (12 mg, 0.010 mmol) was added to the flask and the atmosphere was replaced with argon. After addition of dry toluene (2.0 mL) to the flask using a syringe, the flask was evacuated and filled with argon three times. The mixture was stirred at 120 °C for 24 h and filtered using Celite. The filtrate solvent was distilled off under reduced pressure. The residue was again dissolved in CHCl_3_ and the solution was poured into methanol under vigorous stirring. The precipitated polymer was collected and dried *in vacuo* to afford P2 (40 mg, 34%). *M*_n_ = 4070, *M*_w_/*M*_n_ = 1.80. ^1^H NMR (500 MHz, CDCl_3_) *δ* 7.55–7.38 (m, 6H), 4.28 (m, 4H), 4.08 (m, 4H), 2.71–2.61 (m, 4H), 1.70–1.50 (m, 4H), 1.39–1.16 (m, 12H), 0.90–0.76 (m, 6H); IR (KBr) 2927, 2853, 2360, 1459, 1265, 1220, 1094, 1038, 985, 949, 803 cm^−1^.

P3 (116 mg, 64%), *M*_n_ = 10 100, *M*_w_/*M*_n_ = 1.83; ^1^H NMR (500 MHz, CDCl_3_) *δ* 7.76–7.68 (m, 2H), 7.51 (br s, 2H), 7.48–7.39 (m, 4H), 4.31 (m, 4H), 4.09 (m, 4H), 2.74–2.65 (m, 4H), 2.07–1.95 (m, 4H), 1.70–1.59 (m, 4H), 1.37–1.22 (m, 12H), 1.20–1.01 (m, 24H), 0.87–0.82 (m, 6H), 0.81–0.76 (m, 6H); IR (KBr) 3429, 2935, 2858, 1608, 1547, 1458, 1269, 1038, 984, 949, 818, 721, 698, 579 cm^−1^.

### Synthesis of 3,4-bis(3,3′-dihexyl-2,2′-bithiophen-5-yl)-3-cyclobutene-1,2-dione (7)

To a solution of 6 (84 mg) in CHCl_3_/1,4-dioxane (4/1 (v/v), 1.4 mL), H_2_O (0.11 mL) and TFA (0.22 mL) were added. After stirring at 40 °C for 3 h, the solvent was removed under reduced pressure. The crude material was purified *via* column chromatography on a silica gel (ethyl acetate/hexane = 1/30) to afford 7 (56 mg, 75%) as a red solid: mp 76.3–77.6 °C. ^1^H NMR (500 MHz, CDCl_3_) *δ* 8.30 (s, 2H, thiophene-H), 7.40 (d, *J* = 5.2 Hz, 2H, thiophene-H), 7.01 (d, *J* = 5.2 Hz, 2H, thiophene-H), 2.61 (t, *J* = 7.9 Hz, 4H, thiophene-*CH*_2_CH_2_C_3_H_6_CH_3_), 2.56 (t, *J* = 7.7 Hz, 4H, thiophene-*CH*_2_CH_2_C_3_H_6_CH_3_), 1.64–1.52 (m, 8H, thiophene-CH_2_*CH*_2_C_3_H_6_CH_3_), 1.34–1.17 (m, 24H, thiophene-CH_2_CH_2_*C*_3_*H*_6_CH_3_), 0.85 (t, *J* = 6.9 Hz, 6H, thiophene-CH_2_CH_2_C_3_H_6_*CH*_3_), 0.80 (t, *J* = 7.0 Hz, 6H, thiophene-CH_2_CH_2_C_3_H_6_*CH*_3_); ^13^C NMR (126 MHz, CDCl_3_) *δ* 193.2, 171.9, 144.8, 143.4, 140.7, 135.6, 129.1, 128.6, 126.8, 126.7, 31.55, 31.51, 30.7, 30.6, 29.05, 29.03, 29.01, 28.7, 22.53, 22.52, 14.0; IR (KBr) 3082, 2924, 2854, 1766, 1581, 1458, 1408, 1319, 1200, 1130, 1053, 887, 725 cm^−1^; ESI-MS calcd for C_44_H_59_O_2_S_4_^+^*m*/*z* 747.3392 (M + H)^+^, found *m*/*z* 747.3386.

### Removal of acetal

To a solution of P1 (50 mg, *M*_n_ = 7400, *M*_w_/*M*_n_ = 10.8) in CHCl_3_/1,4-dioxane (4/1 (v/v), 1.4 mL), TsOH·H_2_O (380 mg, 2.0 mmol) was added and the mixture was stirred at 40 °C for 18 h. After CHCl_3_ addition, the solution was washed with H_2_O and dried over anhydrous MgSO_4_. After solvent removal *in vacuo*, the residue was again dissolved in CHCl_3_ and poured into methanol under vigorous stirring. The precipitated polymer was collected and dried *in vacuo* to afford P4 (40 mg, 97%, *M*_n_ = 7200, *M*_w_/*M*_n_ = 11.1). ^1^H NMR (500 MHz, CDCl_3_) *δ* 8.31 (br s, 2H), 2.68–2.18 (m, 4H), 1.62 (m, 4H), 1.25–1.18 (m, 12H), 0.88–0.75 (m, 6H); ^13^C NMR (126 MHz, CDCl_3_) *δ* 192.6, 171.8, 145.8, 137.6, 129.7, 31.4, 30.6, 29.9, 29.0, 22.5, 14.0; IR (KBr) 2931, 2854, 1759, 1573, 1411, 1157, 1038, 852 cm^−1^.

P5 (3.4 mg, 11%, *M*_n_ = 4580, *M*_w_/*M*_n_ = 2.74); ^1^H NMR (500 MHz, CDCl_3_) *δ* 8.33 (br s, 2H), 7.68–7.43 (m, 4H), 2.88–2.46 (m, 4H), 1.78–1.44 (m, 4H), 1.41–1.07 (m, 12H), 0.93–0.68 (m, 6H); IR (KBr) 3435, 2925, 2856, 1764, 1575, 1431, 1385, 1260, 1203, 1098, 1021, 805 cm^−1^.

P6 (27 mg, 33%, *M*_n_ = 11 500, *M*_w_/*M*_n_ = 1.76); ^1^H NMR (500 MHz, CDCl_3_) *δ* 8.33 (br s, 2H), 7.86–7.74 (m, 2H), 7.58–7.44 (m, 4H), 2.87–2.73 (m, 4H), 2.12–1.94 (m, 4H), 1.77–1.54 (m, 4H), 1.41–1.22 (m, 12H), 1.20–0.97 (m, 24H), 0.91–0.82 (m, 6H), 0.81–0.57 (m, 6H); IR (KBr) 3440, 2925, 2853, 1762, 1571, 1532, 1428, 1204, 1099, 821 cm^−1^.

## Results and discussion

### Model reaction for direct Suzuki–Miyaura and Liebeskind–Srogl coupling polymerization of benzene-1,4-diboronic acid and squaric acid dichloride

**Scheme 1:**
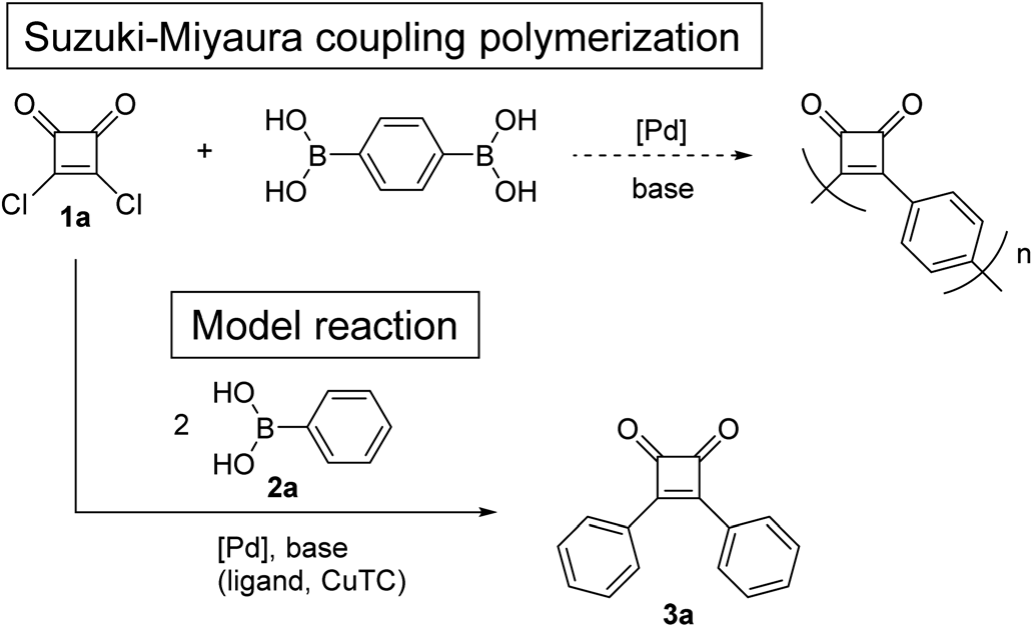
Model reaction for the direct Suzuki–Miyaura coupling polymerization of squaric acid dichloride (1a) and benzene-1,4-diboronic acid.

To investigate whether donor–acceptor π-conjugated polymers can be obtained by direct coupling of squaric acid dichloride (1a) and benzene-1,4-diboronic acid, a model reaction was investigated using 1a and phenylboronic acid (2a) (Scheme 1). The previously reported conditions of Suzuki–Miyaura coupling reaction between acyl chloride and arylboronic acid were used.^18^ First, reaction of 1a and 2a was performed in the presence of 1.0 mol% of Pd(OAc)_2_ and 3.4 equivalents of Na_2_CO_3_ in H_2_O/PEG-2000 at 60 °C.^18*a*^ The ^1^H NMR spectrum of the crude product revealed that 1a was hydrolyzed (Table 1, entry 1). When the coupling reaction was performed in non-aqueous solvent using 2.0 mol% of PdCl_2_(PPh_3_)_2_ and 6.0 equivalents of tri-potassium *n*-hydrate (K_3_PO_4_·*n*H_2_O) in toluene,^18*b*^ the ^1^H NMR spectrum of the crude product indicated that 1a was decomposed by a side reaction other than hydrolysis (entry 2). As Nishihara *et al.* reported that copper(i) thiophene-2-carboxylate (CuTC) promoted the palladium-catalyzed coupling reactions of acid chlorides and arylboronic acids,^19^ the reaction of 1a and 2a was performed under similar conditions, but 1a was decomposed by a side reaction (entry 3).

**Table 1:** Suzuki–Miyaura and Liebeskind–Srogl coupling reactions of 1 and 2^a^

Entry	1 (mmol)	2 (equiv.)	Pd cat. (mol%)	Ligand (mol%)	Base (equiv.)	CuTC (equiv.)	Temp.	Solvent	Yield of 3^b^ (%)
1	1a (0.50)	2a (2.5)	Pd(OAc)_2_ (1.0)	—	Na_2_CO_3_ (3.4)	—	60 °C	H_2_O/PEG-2000 (1.5 g/1.5 g)	0
2	1a (0.50)	2a (2.4)	PdCl_2_(PPh_3_)_2_ (2.0)	—	K_3_PO_4_·*n*H_2_O (6.0)	—	110 °C	Toluene (2.5 mL)	0
3	1a (0.50)	2a (4.0)	Pd(dba)_2_ (5.0)	PPh_3_ (10)	—	1.0	rt	Diethyl ether (15 mL)	0
4	1b (0.20)	2b (2.0)	Pd_2_(dba)_3_ (3.5)	TFP (9)	—	2.5	55 °C	THF (10 mL)	56
5	1b (0.20)	2b (2.0)	Pd(PPh_3_)_4_ (3.5)	—	—	2.5	55 °C	THF (10 mL)	44
6	1b (0.20)	2b (2.0)	Pd(OAc)_2_ (3.5)	SPhos (10)	—	2.5	55 °C	THF (10 mL)	0
7	1b (0.20)	2b (2.0)	Pd_2_(dba)_3_ (3.5)	TFP (9)	—	5.0	55 °C	THF (10 mL)	45
8	1b (0.20)	2b (2.0)	Pd_2_(dba)_3_ (7.0)	TFP (9)	—	2.5	55 °C	THF (10 mL)	63

a The reaction of 1 and 2 was performed as indicated.

b Yield was calculated from the ^1^H NMR spectra of the products after column chromatography.

**Scheme 2:**
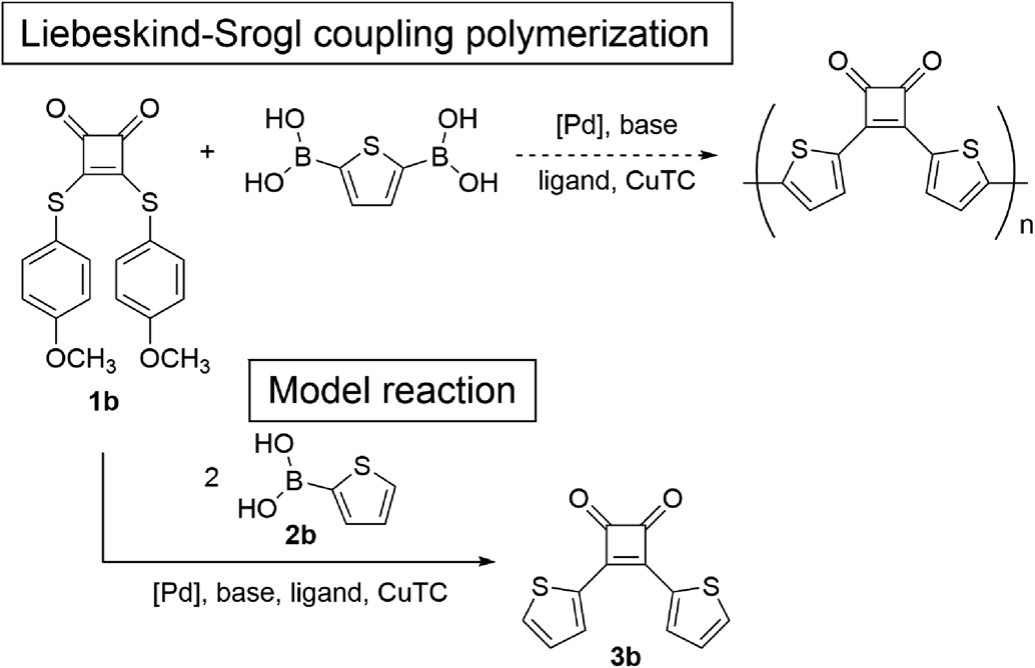
Model reaction for the direct Liebeskind–Srogl coupling polymerization of 3,4-bis[(4-methoxyphenyl)thio]-3-cyclobutene-1,2-dione (1b) and 2,5-thiophenediboronic acid.

Next, we focused on the Liebeskind–Srogl coupling reaction^20^ using bisarylthiocyclobutenedione 1b and 2-thiopheneboronic acid (2b) in the presence of CuTC and a palladium catalyst as a model reaction of the polymerization of 1b and 2,5-thiophenediboronic acid (Scheme 2). Because the Peña-Cabrera group reported that the Liebeskind–Srogl coupling of 1b and 3-thiopheneboronic acid (the regioisomer of 2b) affords the desired product in 94% yield,^17^ the reaction of 1b and 2b was performed under similar conditions using 2.5 equivalents of CuTC, 9 mol% of tri(2-furyl)phosphine (TFP), and 3.5 mol% of Pd_2_(dba)_3_ in THF at 55 °C. The reaction for 19 h afforded 3b in 56% yield (entry 4). When Pd(PPh_3_)_4_ was used instead of Pd_2_(dba)_3_, the yields of 3b were not improved (entry 5). In addition, using Pd(OAc)_2_/SPhos resulted in no reaction (entry 6). Based on the conditions used for entry 4, increasing CuTC did not increase the yields (entry 7). When an increased amount of Pd_2_(dba)_3_ (7.0 mol%) was used, the yield of 3b improved to 63% (entry 8), but because the model reaction did not proceed quantitatively, the Liebeskind–Srogl coupling reaction was difficult to apply to polymerization.

### Synthesis and Kumada–Tamao–Corriu coupling polymerization of the acetal monomer

**Scheme 3:**
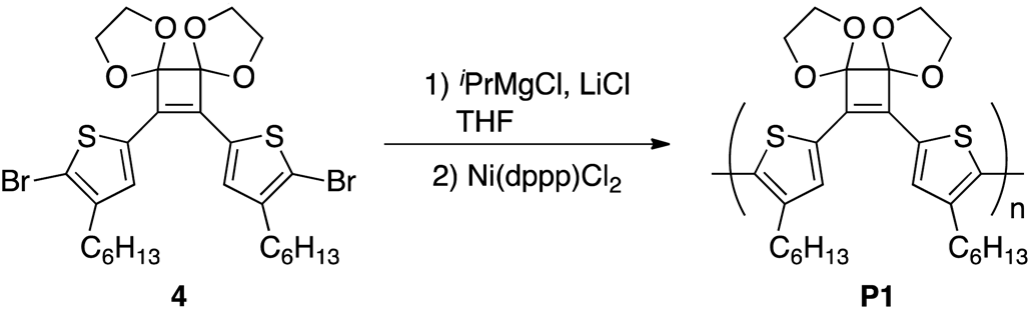
Polymerization of 4*via* Kumada–Tamao–Corriu coupling.

Introducing various polymerizable functional groups at the 2- and 5-positions of the 3-alkylthiophene is relatively facile and the synthesis of π-conjugated polymers composed of alkylthiophene has been achieved by various coupling reactions.^21^ For the π-conjugated polymers containing alkylthiophene and cyclobutenedione units, Huang *et al.* reported Suzuki–Miyaura^10*a*^ and Stille coupling polymerizations^10*b*^ using 3,4-bis(5-bromo-4-hexylthiophen-2-yl)-3-cyclobutene-1,2-dione as a monomer. However, only the two articles have reported the synthesis of π-conjugated cyclobutenedione polymers by coupling reactions likely because of the high susceptibility of the cyclobutenedione unit to nucleophiles and bases, limiting the available polymerization methods. We thought that the yields of the model reactions described in the previous section did not improved for the same reason. Therefore, to suppress side reactions at the cyclobutenedione units induced by bases and nucleophiles, an acetal-protected cyclobutenedione monomer was used. For the synthesis of π-conjugated polymers with carbonyl groups by coupling reaction, the Jenekhe and Jia groups reported the synthesis of poly(3-alkanoylthiophene) *via* Kumada–Tamao–Corriu coupling polymerization of a thiophene monomer whose acyl group was protected as an acetal.^22^ Thus, the acetal groups are compatible with Grignard reagents. In addition, acetal groups can be easily hydrolyzed under aqueous acidic conditions. Therefore, we tried to synthesize the polymer P1 by Kumada–Tamao–Corriu coupling polymerization of the acetal monomer 4 (Scheme 3). The synthesis of monomer 4 was described in the ESI (Scheme S1†).

**Fig. 1:**
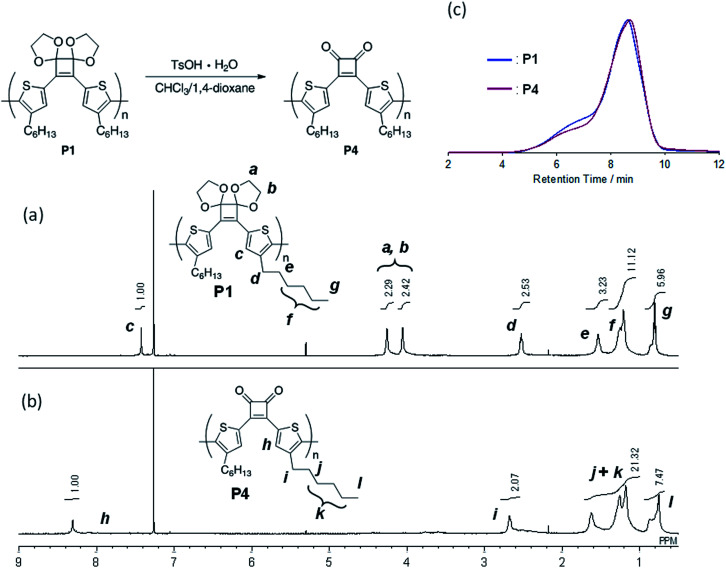
^1^H NMR spectra (500 MHz, CDCl_3_) of (a) P1 and (b) P4, and (c) GPC profiles (eluent: CHCl_3_) of P1 and P4.

For polymerization, 4 was reacted with an equimolar amount of ^i^PrMgCl in the presence of 1.3 equivalents of LiCl in THF at 0 °C. Subsequently, 4 mol% Ni(dppp)Cl_2_ was added and the reaction was performed at 40 °C for 24 h. After purification, the polymer P1 with *M*_n_ and *M*_w_ values of 4500 and 11 800, respectively, was obtained in 44% yield (Table 2, entry 1). To perform the bromine–magnesium exchange reaction under milder conditions, 4 was reacted with ^i^PrMgCl at −20 °C. After Ni catalyst addition and polymerization at 40 °C, P1 with slightly increased *M*_n_ and *M*_w_ values was obtained (entry 2). Increasing or decreasing the Ni catalyst loading to 8 mol% and 2 mol%, respectively, decreased the molecular weights of P1 (entries 3 and 4). When the polymerization time was extended to 8 h using 4 equivalents of the Ni catalyst, P1 with the highest *M*_n_ and *M*_w_ values was obtained in a good yield (entry 5). The obtained polymer showed signals derived from the acetal group at 4.6–4.2 ppm in the ^1^H NMR spectrum (Fig. 1a) and from the cyclobutene ring in the ^13^C NMR spectrum (Fig. S1†). These results demonstrated that conjugated polymers containing a cyclobutene ring can be synthesized under Kumada–Tamao–Corriu coupling conditions using an acetal-protecting strategy.

**Table 2:** Polymerization of monomer 4^a^

Entry	Temp^b^ (°C)	Ni(dppp)Cl_2_ (mol%)	Time^c^ (h)	*M*_n_ d	*M*_w_ d	Yield (%)
1	0	4	24	4500	11 800	44
2	−20	4	24	4700	15 700	22^e^
3	−20	8	24	1100	5700	49
4	−20	2	24	4100	10 000	44
5	−20	4	48	7400	80 100	76

a Monomer 4 was initially reacted with ^i^PrMgCl (1.0 equiv.) in the presence of LiCl (1.3 equiv.) in THF for 1 h. After Ni(dppp)Cl_2_ addition, the reaction was performed at 40 °C.

b The temperature for the reaction of 4 with ^i^PrMgCl in the presence of LiCl in THF for 1 h.

c Reaction time after Ni(dppp)Cl_2_ addition.

d Determined by GPC based on PSt standards (eluent: CHCl_3_).

e The purification procedure was performed twice.

### Suzuki–Miyaura coupling of the acetal monomer

To expand the scope of the acetal monomer polymerization, the model reaction of Suzuki–Miyaura coupling polymerization was performed using 1 equivalent of 4 and 2.2 equivalents of 3-hexylthiophene-2-boronic acid pinacol ester in the presence of 5 mol% of Pd(PPh_3_)_4_ and 4.4 equivalents of Cs_2_CO_3_ in toluene at 120 °C (Scheme S2†).^23^ This reaction afforded the target model compound in good yield (83%).

Next, to conduct Suzuki–Miyaura coupling polymerization, the introduction of boronic acid pinacol ester (Bpin) to 4 was examined. 4 was reacted with *n*-BuLi in THF at −78 °C, then 4,4,5,5-tetramethyl-1,3,2-dioxaborolane was added and the reaction was carried out at room temperature for 22 h.^24^ The ^1^H NMR spectrum of the crude product showed that Bpin was introduced to 4 but the product could not be separated from the by-products *via* silica gel column chromatography. Similarly, after the reaction of *n*-BuLi with 4 in THF at −78 °C, 2-isopropoxy-4,4,5,5-tetramethyl-1,3,2-dioxaborolane or triisopropyl borate was added.^25,26^ However, the ^1^H NMR spectrum of the crude products showed small signals of 4 and those corresponding to many by-products. Further, 4 (1.0 equivalent) and bis(pinacolato)diboron (4.0 equivalents) were reacted in the presence of PdCl_2_(dppf) (0.20 equivalents) and potassium acetate (6.0 equivalents) in 1,4-dioxane at 80 °C for 22 h, but the desired product was not obtained.

**Scheme 4:**
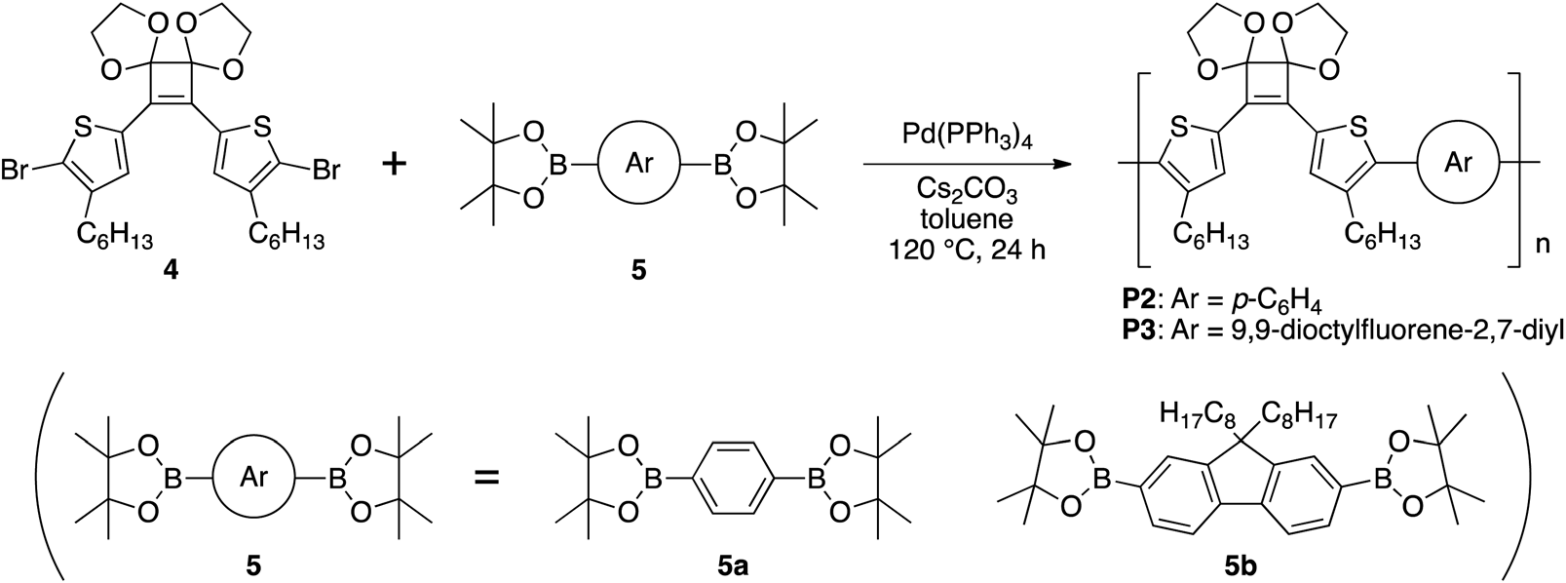
Polymerization of 4 and 5 under Suzuki–Miyaura coupling conditions.

Therefore, to synthesize a polymer by Suzuki–Miyaura coupling, 4 was polymerized with commercially available bis(boronic acid pinacol ester) monomers. 1,4-Phenylenediboronic acid pinacol ester (5a) and 9,9-dioctylfluorene-2,7-diboronic acid pinacol ester (5b) were used as monomers because their polymerization with 4 should afford polymers with different conjugation lengths. The monomer 4 was reacted with an equal amounts of 5a or 5b in the presence of 5 mol% of Pd(PPh_3_)_4_ and 4.4 equivalents of Cs_2_CO_3_ in toluene at 120 °C for 24 h (Scheme 4). GPC elution curves of the crude products showed peaks corresponding to P2 (*M*_n_ = 4070, *M*_w_/*M*_n_ = 1.80) and P3 (*M*_n_ = 10 100, *M*_w_/*M*_n_ = 1.83) and the ^1^H NMR spectra also agreed with the corresponding polymers (Fig. S3b and S4b†). Therefore, the target polymers (P2 and P3) were obtained under the same conditions as those used for the model reaction.

### Hydrolysis of the acetal groups

**Fig. 2:**
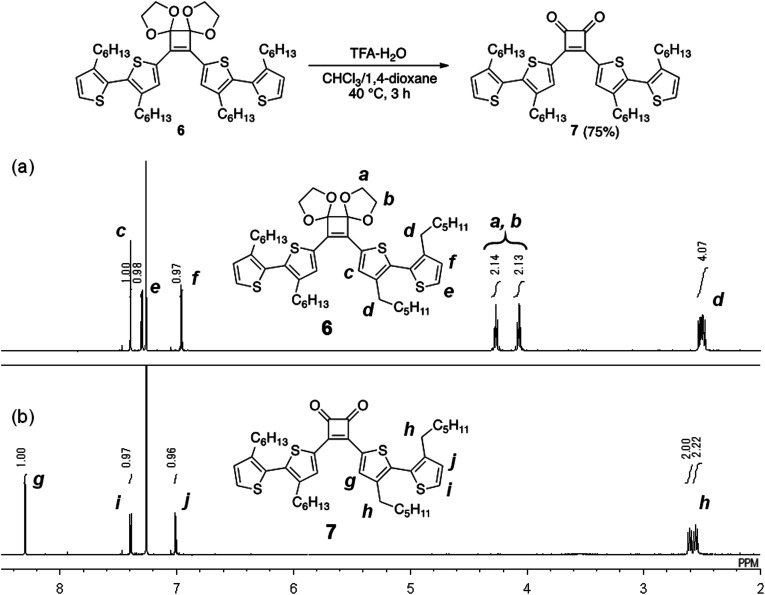
^1^H NMR spectra (500 MHz, CDCl_3_) of (a) 6 and (b) 7.

Initially, as a model reaction, the hydrolysis of the acetal groups of 6 was investigated under aqueous acidic conditions (Fig. 2).^22^ When 6 was treated with trifluoroacetic acid (TFA; 29 equivalents) and distilled water (61 equivalents) in a mixed solvent of CHCl_3_/1,4-dioxane (4/1, v/v) at 40 °C for 3 h, the ^1^H NMR spectrum of the product corresponded to target compound 7 and acetal signals were not detected (Fig. 2). Therefore, the acetal was easily hydrolyzed under acidic conditions without side reactions, affording 7 in good yield (75%). However, acetal hydrolysis of P1 under the same conditions resulted in precipitate formation as the reaction progressed and the ^1^H NMR spectrum of the crude product showed residual acetal signals.

**Scheme 5:**
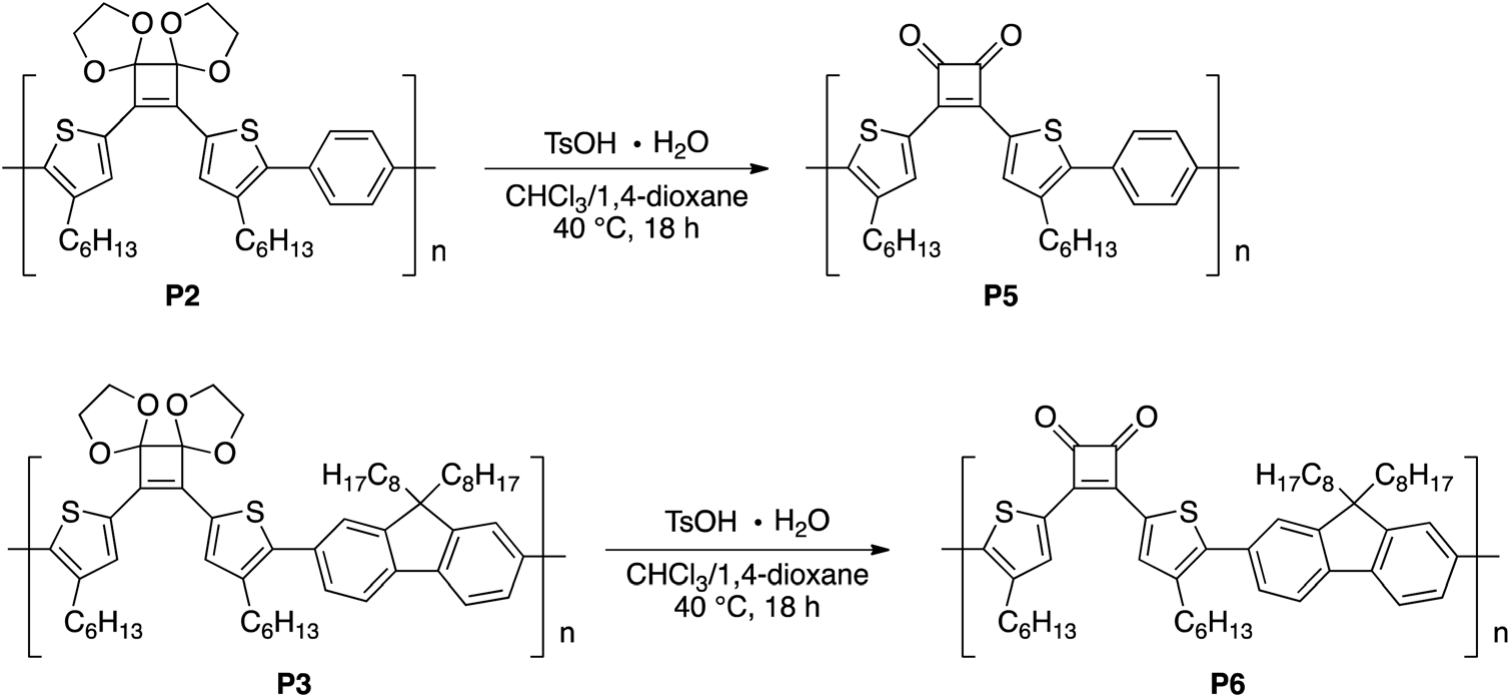
Hydrolysis of acetal groups in P2 and P3.

Next, hydrolysis using *p*-toluenesulfonic acid monohydrate (TsOH·H_2_O; 20 equivalents to the repeating unit) was performed in a mixed solvent of CHCl_3_/1,4-dioxane (4/1, v/v) at 40 °C for 18 h. Deprotection of P1 proceeded homogeneously and the product was purified by precipitation in a large excess of methanol. The GPC elution curve of the deprotected polymer P4 shifted slightly toward lower molecular weight region (*M*_n_ = 7200, *M*_w_/*M*_n_ = 11.1) compared to P1 (*M*_n_ = 7400, *M*_w_/*M*_n_ = 10.8; Fig. 1c). Furthermore, the ^1^H NMR spectrum of the products P4 did not show acetal-methylene protons at approximately 4.6–4.2 ppm (Fig. 1b) and the ^13^C NMR spectrum contained signals derived from the cyclobutenedione ring (Fig. S2†). Therefore, the acetal groups were removed without polymer decomposition and P4 containing cyclobutenedione rings in the main chain was obtained in high yield (97%). This demonstrates that the use of an acid hydrate in non-aqueous solvents enabled hydrolysis of the acetal group without polymer precipitation. Hydrolysis of the acetal groups in P2 and P3 under the same conditions afforded P5 (11%) and P6 (33%), respectively (Scheme 5, Fig. S3c and S4c†). The low yields of P5 and P6 are not due to side reactions and can instead be attributed to a large loss of the products during precipitation purification.

### Optical properties of the π-conjugated cyclobutenedione polymers

**Fig. 3:**
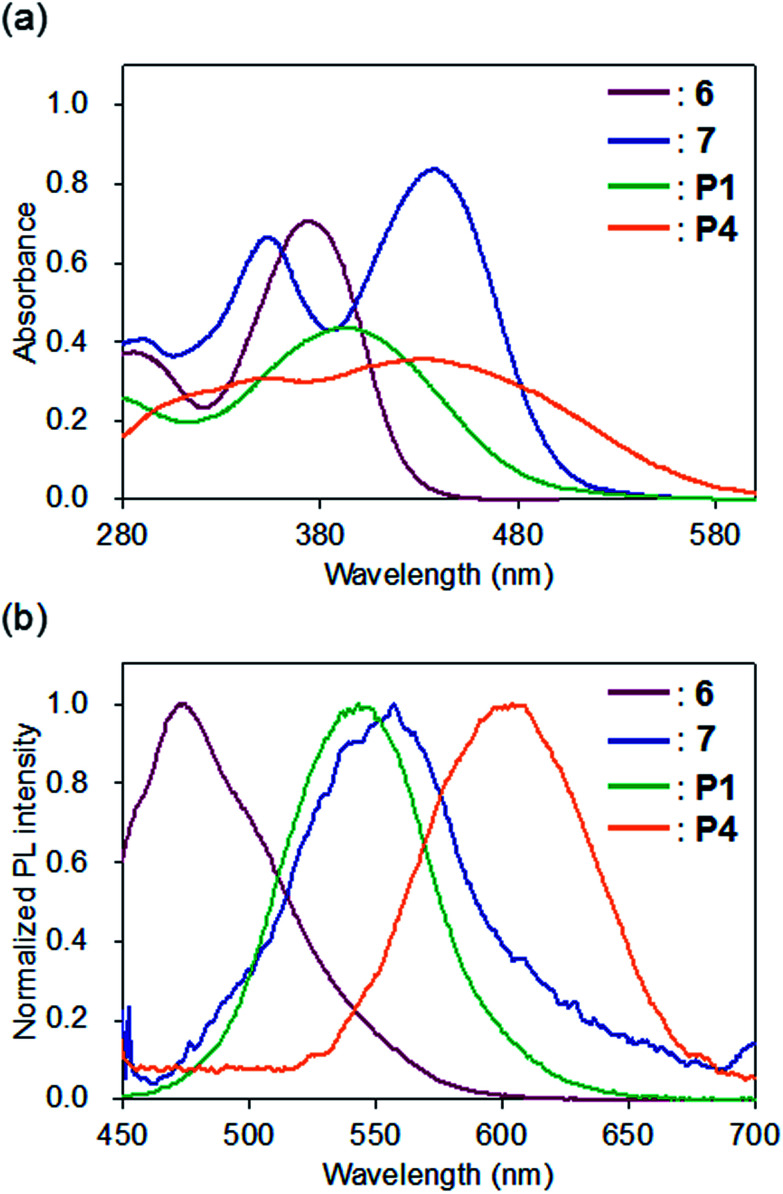
(a) UV-vis absorption and (b) photoluminescence spectra of 6 (red), 7 (blue), P1 (green), and P4 (orange) in CHCl_3_ (1.0 × 10^−5^ M); PL excitation at the longest absorption maximum wavelength.

The optical properties of P1 and P4, which have shorter conjugation lengths than P2, P3, P5, and P6, were compared with model compounds 6 and 7 (Fig. 3). The UV-vis spectra of 6 and P1 in CHCl_3_ showed unimodal peaks at maximum absorption wavelengths (*λ*_abs_) of 374 and 393 nm, respectively, whereas 7 (435 and 353 nm) and P4 (433 and 340 nm) exhibited bimodal absorption peaks (Fig. 3a). The *λ*_abs_ of P1 shifted to 19 nm longer than 6, whereas that of P4 was shorter than 7. The bimodal UV-vis absorption curves of 7 and P4 are consistent with previously reported results showing donor–acceptor-type π-conjugated polymers containing cyclobutenedione units exhibited two absorption bands.^10*a*^ The absorption peak at the shorter wavelength side seems to arise from the π–π* transition of the polymer repeating units, and the absorption peak on the longer wavelength side will be due to an intramolecular charge transfer transition between the donor and the acceptor units. In the spectrum of P4, a broad absorption band was observed at a longer wavelength (approximately 480 nm), which was not observed for 7. This indicates that the conjugation length of P4 is longer than that of 7.

In contrast, the *λ*_abs_ of the acetal-protected polymers, P2 and P3, and deprotected polymers P5 and P6 in CHCl_3_ were 394, 407, 443 and 473 nm, respectively (Fig. S5a and S5b†). The *λ*_abs_ of P2 and P5 were observed at similar wavelengths compared to P1 and P4, respectively, suggesting that conjugation length did not increase when a benzene ring was introduced into the monomer unit of the polymer main chain. In contrast, the longer *λ*_abs_ of P3 and P6 compared to P1 and P4, respectively, indicates that the polymer conjugation length increased upon introduction of a fluorene ring to the monomer unit.

**Fig. 4:**
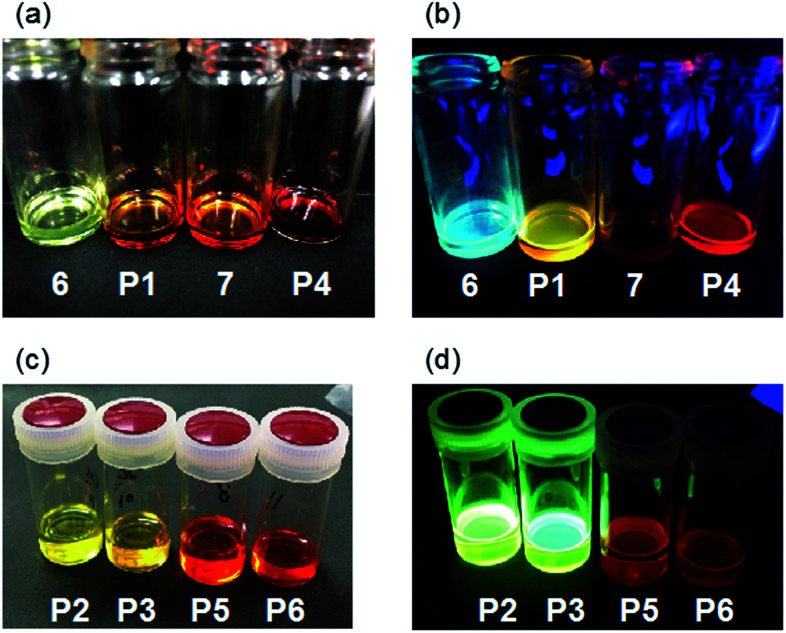
Pictures of 6, 7, and P1–P6 (a and c) under fluorescent lamp irradiation and (b and d) under UV lamp irradiation (365 nm).

In the fluorescence spectra, the emission maximum wavelengths of 6, 7, P1, and P4 in CHCl_3_ were observed at 473, 557, 543, and 604 nm, respectively (Fig. 3b). Compared to the model compound and polymer with the same cyclobutenedione unit (6*vs.*P1; 7*vs.*P4), the emission maximum wavelengths of the prepared polymers were longer than those of the model compounds. In both cases of the model compounds (6*vs.*7) and the polymers (P1*vs.*P4), removal of acetal groups red-shifted the emission maximum wavelengths. This was also observed for other polymers, and the emission maxima of the deprotected polymers P5 (*λ*_em_ = 602 nm) and P6 (*λ*_em_ = 559 nm) were longer than those of the corresponding acetal polymers P2 (*λ*_em_ = 546 nm) and P3 (*λ*_em_ = 491 nm; Fig. S5c and S5d†). When the chloroform solutions of the model compounds and polymers were irradiated using a UV lamp (365 nm, 4 W), 6, P1, and P4 emitted blue, yellowish, and red light, respectively, but the solution of 7 was non-emissive (Fig. 4b). In addition, the solutions of P2 and P3 emitted yellowish-green light and those of P5 and P6 emitted red light (Fig. 4d). It is unclear the reason why the solution of 7 did not emit, but these results demonstrated that the luminescence character depends on the electronic states (acetal-protected *vs.* deprotected) of the cyclobutene unit and on the molecular weight (small molecule *vs.* polymer). Furthermore, absorption and fluorescence peaks of polymers P4–P6 were longer than those of previously reported cyclobutenedione conjugated polymers.^9,10^ These results indicate that acetal-protecting strategy is a useful method for synthesizing conjugated polymers with a high degree of polymerization and significant conjugation length.

## Conclusions

In summary, the synthesis of donor–acceptor-type π-conjugated polymers consisting of cyclobutenedione as an acceptor and bithiophene as a donor was developed. When the carbonyl groups of cyclobutenedione were protected as acetals, the reaction proceeded under Kumada–Tamao–Corriu coupling conditions using Grignard reagents. Furthermore, the π-conjugated cyclobutenedione polymers whose repeating units exhibited different conjugation lengths were synthesized by Suzuki–Miyaura coupling reaction. The acetal groups were smoothly removed under acidic conditions. The absorption and fluorescence spectra of the prepared polymers demonstrated that acetal removal red-shifted the absorption and emission maximum wavelengths and generated bimodal peaks likely due to intramolecular donor–acceptor interaction. A synthetic study of the cyclobutenedione polymers with other π-conjugated lengths is currently underway.

## Conflicts of interest

There are no conflicts to declare.

## Supplementary Material

RA-009-C9RA08275A-s001

## References

[cit1] Wurm F. R., Klok H.-A. (2013). Chem. Soc. Rev..

[cit2] Prohens R., Martorell G., Ballester P., Costa A. (2001). Chem. Commun..

[cit3] Alemán J., Parra A., Jiang H., Jørgensen K. A. (2011). Chem.–Eur. J..

[cit4] Liebeskind L. S., Iyer S., Jewell Jr C. F. (1986). J. Org. Chem..

[cit5] Rubin Y., Knobler C. B., Diederich F. (1990). J. Am. Chem. Soc..

[cit6] Krayushkin M. M., Shirinian V. Z., Belen'kii L. I., Shadronov A. Y., Martynkin A. Y., Uzhinov B. M. (2002). Mendeleev Commun..

[cit7] Liu Z.-Y., Wang Y.-M., Han Y.-X., Liu L., Jin J., Yi H., Li Z.-R., Jiang J.-D., Boykin D. W. (2013). Eur. J. Med. Chem..

[cit8] Imai Y., Shiratori M., Inoue T., Kakimoto M. (2002). J. Polym. Sci., Part A: Polym. Chem..

[cit9] Jeong J. K., Choi S. J., Rhee T. H., Yang N. C., Suh D. H. (1999). Polym. Bull..

[cit10] Huo E. F., Zou Y., Sun H. Q., Huang Y., Lu Z. Y., Jiang Q. (2011). Chin. Chem. Lett..

[cit11] Yang N. C., Suh D. H. (2001). Macromol. Rapid Commun..

[cit12] Maier G., Rohr C. (1996). Liebigs Ann..

[cit13] Allen A. D., Ma J., McAllister M. A., Tidwell T. T., Zhao D.-c. (1995). Acc. Chem. Res..

[cit14] Wang Z. Y., Suzzarini L. (1996). Macromolecules.

[cit15] Kraus J. L. (1985). Tetrahedron Lett..

[cit16] Malicka J. M., Sandeep A., Monti F., Bandini E., Gazzano M., Ranjith C., Praveen V. K., Ajayaghosh A., Armaroli N. (2013). Chem.–Eur. J..

[cit17] Aguilar-Aguilar A., Liebeskind L. S., Peña-Cabrera E. (2007). J. Org. Chem..

[cit18] Xin B., Zhang Y., Cheng K. (2006). J. Org. Chem..

[cit19] Nishihara Y., Inoue Y., Fujisawa M., Takagi K. (2005). Synlett.

[cit20] Liebeskind L. S., Srogl J. (2000). J. Am. Chem. Soc..

[cit21] Dai C.-A., Yen W.-C., Lee Y.-H., Ho C.-C., Su W.-F. (2007). J. Am. Chem. Soc..

[cit22] Wang C., Kim F. S., Ren G., Xu Y., Pang Y., Jenekhe S. A., Jia L. (2010). J. Polym. Sci., Part A: Polym. Chem..

[cit23] Hermerschmidt F., Kalogirou A. S., Min J., Zissimou G. A., Tuladhar S. M., Ameri T., Faber H., Itskos G., Choulis S. A., Anthopoulos T. D., Bradley D. D. C., Nelson J., Brabec C. J., Koutentis P. A. (2015). J. Mater. Chem. C.

[cit24] Li J., Yan M., Xie Y., Qiao Q. (2011). Energy Environ. Sci..

[cit25] Fernando R., Mao Z., Sauvé G. (2013). Org. Electron..

[cit26] Adachi K., Hirao H., Matsumoto K., Kubo T. (2014). Org. Lett..

