# Supplementary material for: Synthesis and characterization of cyclobutenedione–bithiophene π-conjugated polymers: acetal-protecting strategy for Kumada–Tamao–Corriu coupling polymerization between aryl bromide and Grignard reagents
Source: RSC Adv. 2019 Dec 11;9(70):40863–72. doi: 10.1039/c9ra08275a (PMC9076255; doi:10.1039/c9ra08275a)

Electronic Supplementary Material (ESI) for RSC Advances.
This journal is © The Royal Society of Chemistry 2019

Synthesis and Characterization of Cyclobutenedione-Bithiophene π-Conjugated Polymers: Acetal-Protecting Strategy for Kumada-Tamao-Corriu Coupling Polymerization Between Aryl Bromide and Grignard Reagents

Tomoyuki Ohishi,* Takuma Sone, Kohei Oda, Akihiro Yokoyama*

Department of Materials and Life Science, Faculty of Science and Technology, Seikei University, 3-3-1 Kichijoji-kitamachi, Musashino, Tokyo 180-8633, Japan

* Corresponding authors

E-mail: t-ohishi@st.seikei.ac.jp (T. Ohishi), ayokoyama@st.seikei.ac.jp (A. Yokoyama)

Materials

Trimethylsilyl trifluoromethanesulfonate (TfOTMS; TCI), 1,2-bis[(trimethylsilyl)oxy]ethane (TCI), 3-hexylthiophene-2-boronic acid pinacol ester (Aldrich), and dehydrated pyridine (Wako) were used as-received without purification. 2-Bromo-3-hexylthiophene[1] was synthesized according to procedures described in the literature.

Synthesis of the monomer

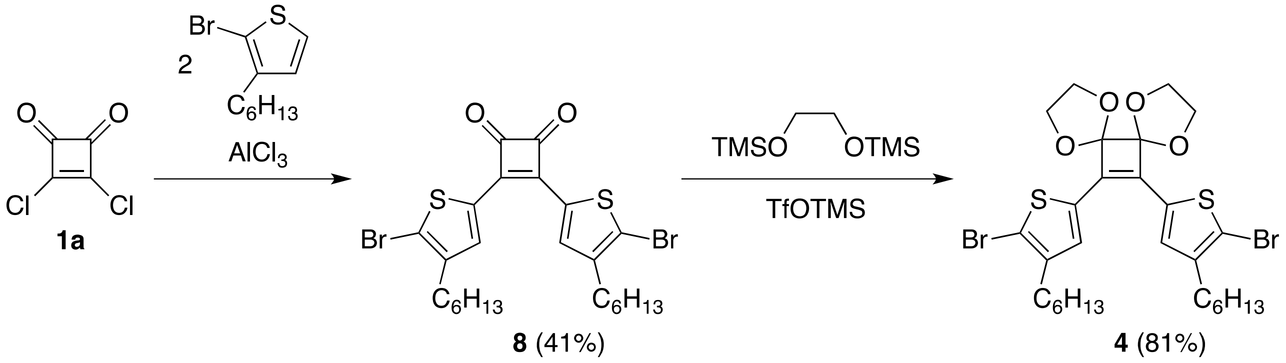

Scheme S1. Synthesis of monomer **4**.

Synthesis of 3,4-bis(5-bromo-4-hexylthiophen-2-yl)-3-cyclobutene-1,2-dione (8)[2]

A two-necked round-bottomed flask equipped with a three-way stopcock and a rubber septum was heated under reduced pressure and subsequently cooled to room temperature under an argon atmosphere. $AlCl_3$ (1.85 g, 13.9 mmol) and dry CH_2Cl_2 (20 mL) were added to the flask, which was evacuated and filled with argon three times. The flask was cooled to –20 °C and a solution of **1a** (1.00 g, 6.60 mmol) in dry CH_2Cl_2 (10 mL) and a solution of 2-bromo-3-hexylthiophene (1.65 g, 6.65 mmol) in dry CH_2Cl_2 (5.0 mL) were added dropwise successively to the flask. After the mixture was stirred at –20 °C for 3 h, a solution of 2-bromo-3-hexylthiophene (1.65 g, 6.65 mmol) in dry CH_2Cl_2 (5.0 mL) and dry pyridine (0.5 mL) was added dropwise. Then the reactant was warmed to 0 °C and

after 19 h of stirring, the reaction mixture was poured into ice water and extracted with CH_2Cl_2. The organic layer was successively washed with saturated aqueous $NaHCO_3$ and water then dried over anhydrous $MgSO_4$. After evaporation of the solvent, the crude product was purified via silica gel column chromatography (hexane/CH_2Cl_2 = 1/1, v/v) and recrystallization from acetonitrile to yield 1.54 g of **8** as a brown solid (41%). ^{1}H NMR (500 MHz, $CDCl_3$) δ 8.06 (s, 2 H, thiophene-H), 2.68 (t, J = 7.7 Hz, 4 H, thiophene-$CH_2CH_2C_3H_6CH_3$), 1.65 (quint, J = 7.6 Hz, 4 H, thiophene-$CH_2CH_2C_3H_6CH_3$), 1.42–1.28 (m, 12 H, thiophene-$CH_2CH_2C_3H_6CH_3$), 0.90 (t, J = 7.0 Hz, 6 H, thiophene-$CH_2CH_2C_3H_6CH_3$).

Synthesis of 3,4-bis(5-bromo-4-hexylthiophen-2-yl)-3-cyclobutenedione bis(ethylene ketal) (4)[3]

A round-bottomed flask equipped with a three-way stopcock was heated under reduced pressure and subsequently cooled to room temperature under an argon atmosphere. Then, **8** (50 mg, 0.088 mmol), 1,2-bis[(trimethylsilyl)oxy]ethane (1.3 mL, 5.0 mmol), and TfOTMS (0.10 mL, 0.60 mmol) were added to the flask under an argon atmosphere. The mixture was stirred at 80 °C for 30 min. After cooling to 0 °C, dry pyridine (0.15 mL, 1.8 mmol) was added. The mixture was filtered, and the solid was washed with diethyl ether. After removal of the solvent from the filtrate, the crude product was chromatographed on triethylamine-treated silica gel (hexane/ethyl acetate = 5/1, v/v) to yield 47 mg of **4** as a yellow oil (81%). ^{1}H NMR (500 MHz, $CDCl_3$) δ 7.15 (s, 2 H, thiophene-H), 4.26–4.20 (m, 4 H, OCH_2CH_2O), 4.07–4.00 (m, 4 H, OCH_2CH_2O), 2.56 (t, J = 7.7 Hz, 4 H, thiophene-$CH_2CH_2C_3H_6CH_3$), 1.58 (quint, J = 7.2 Hz, 4 H, thiophene-$CH_2CH_2C_3H_6CH_3$), 1.39–1.25 (m, 12 H, thiophene-$CH_2CH_2C_3H_6CH_3$), 0.89 (t, J = 6.9 Hz, 6 H, thiophene-$CH_2CH_2C_3H_6CH_3$); ^{13}C NMR (126 MHz, $CDCl_3$) δ 142.6, 132.9, 131.3, 129.5, 114.0, 112.2, 65.8, 31.6, 29.6, 29.3, 28.8, 22.6, 14.1; IR (KBr) 2926, 2861, 1727, 1532, 1454, 1369, 1274, 1126, 1033, 984, 951, 837, 797, 727, 633, 576 cm^{-1}; ESI-MS calcd for $C_{28}H_{37}{}^{79}Br^{81}BrO_4S_2^+$ m/z 661.0474 (M+H)$^+$, found m/z 661.0477.

Synthesis of 3,4-bis(3,3'-dihexyl-2,2'-bithiophen-5-yl)-3-cyclobutenedione bis(ethylene ketal) (6)[4]

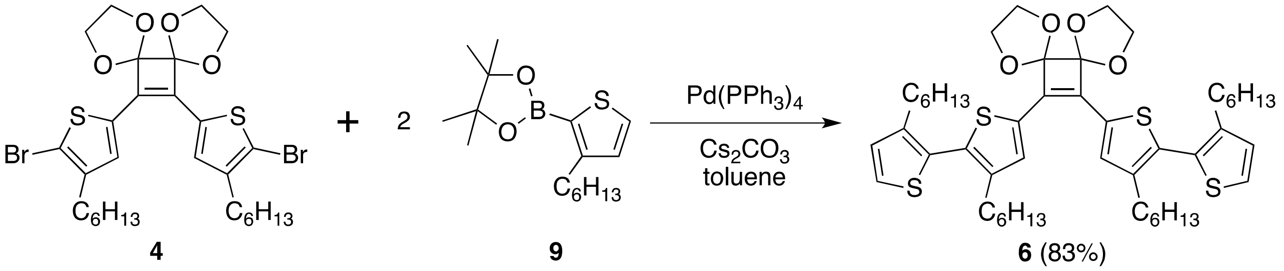

Scheme S2. Suzuki-Miyaura coupling reaction of **4** with 3-hexylthiophene-2-boronic acid pinacol ester (**9**).

A round-bottomed flask equipped with a three-way stopcock was heated under reduced pressure and

subsequently cooled to room temperature under an argon atmosphere. Then, **4** (132 mg, 0.200 mmol), 3-hexylthiophene-2-boronic acid pinacol ester (**9**) (132 mg, 0.440 mmol), and Cs_2CO_3 (286 mg, 0.880 mmol) were added to the flask, which was evacuated and filled with argon three times. $Pd(PPh_3)_4$ (12 mg, 0.010 mmol) was added to the flask and the atmosphere was replaced with argon. After addition of dry toluene (2.0 mL) to the flask using a syringe, the flask was evacuated and filled with argon three times. The mixture was stirred at 120 °C for 16 h and filtered using Celite. The filtrate solvent was distilled off under reduced pressure and the crude product was chromatographed on a triethylamine-treated silica gel (hexane/ethyl acetate = 4/1, v/v) to yield 138 mg of **6** as a yellow oil (83%). ^{1}H NMR (500 MHz, $CDCl_3$) δ 7.40 (s, 2 H, thiophene-H), 7.30 (d, *J* = 5.4 Hz, 2 H, thiophene-H), 6.96 (d, *J* = 5.2 Hz, 2 H, thiophene-H), 4.30–4.24 (m, 4 H, O$CH_2$$CH_2$O), 4.10–4.04 (m, 4 H, O$CH_2$$CH_2$O), 2.53–2.48 (m, 8 H, thiophene-$CH_2CH_2C_3H_6CH_3$), 1.53 (quint, *J* = 8.1 Hz, 8 H, thiophene-$CH_2CH_2C_3H_6CH_3$), 1.28–1.17 (m, 24 H, thiophene-$CH_2CH_2C_3H_6CH_3$), 0.85–0.82 (m, 12 H, thiophene-$CH_2CH_2C_3H_6CH_3$); ^{13}C NMR (126 MHz, $CDCl_3$) δ 142.7, 142.6, 132.9, 131.5, 131.4, 130.3, 128.6, 128.1, 125.6, 114.1, 65.9, 31.64, 31.61, 30.8, 30.6, 29.1, 29.0, 28.9, 28.7, 22.6, 14.1; IR (KBr) 3097, 2927, 2858, 1466, 1423, 1273, 1223, 1038, 984, 949, 837, 741 cm^{-1}; ESI-MS calcd for $C_{48}H_{67}O_4S_4^+$ m/z 835.3917 (M+H)$^+$, found m/z 835.3907.

1. F. P. V. Koch, P. Smith, M. Heeney, *J. Am. Chem. Soc.*, 2013, **135**, 13695–13698.
2. E. F. Huo, Y. Zou, H. Q. Sun, Y. Huang, Z. Y. Lu, Q. Jiang, *Chin. Chem. Lett.*, 2011, **22**, 1326–1330
3. J. Kuhni, P. Belser, *Org. Lett.*, 2007, **9**, 1915–1918.
4. F. Hermerschmidt, A. S. Kalogirou, J. Min, G. A. Zissimou, S. M. Tuladhar, T. Ameri, H. Faber, G. Itskos, S. A. Choulis, T. D. Anthopoulos, D. D. C. Bradley, J. Nelson, C. J. Brabec, P. A. Koutentis, *J. Mater. Chem. C.*, 2015, **3**, 2358–2365.

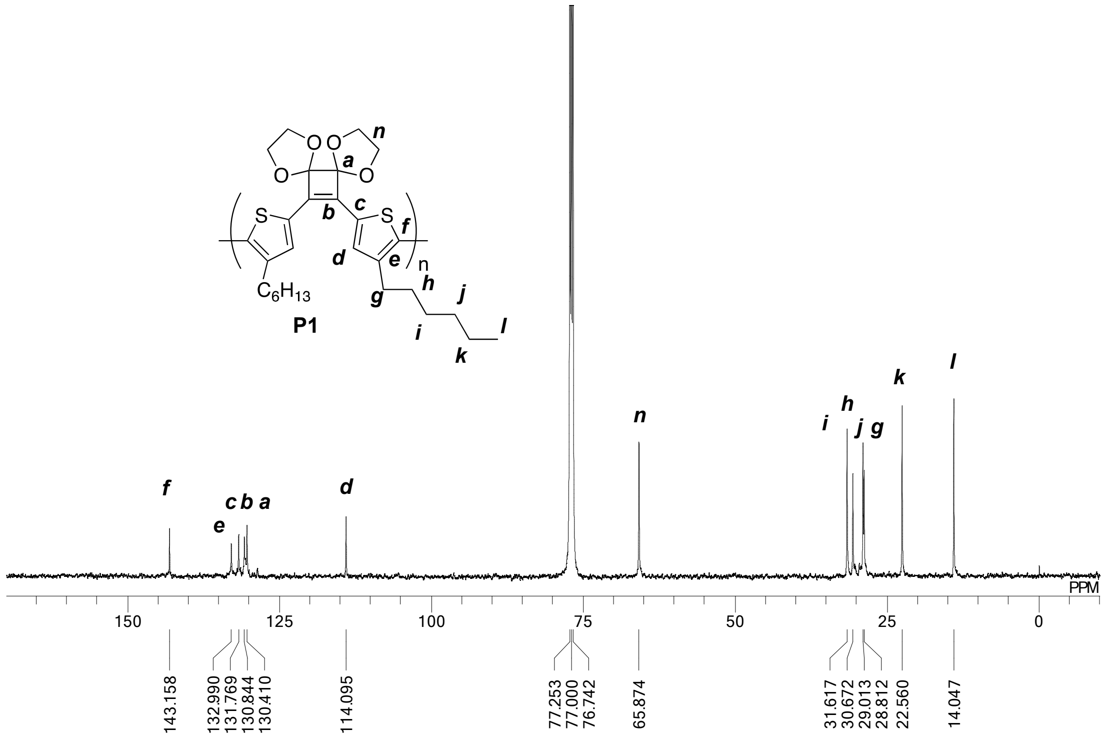

Figure S1. ^{13}C NMR spectrum (126 MHz, $CDCl_3$) of **P1**.

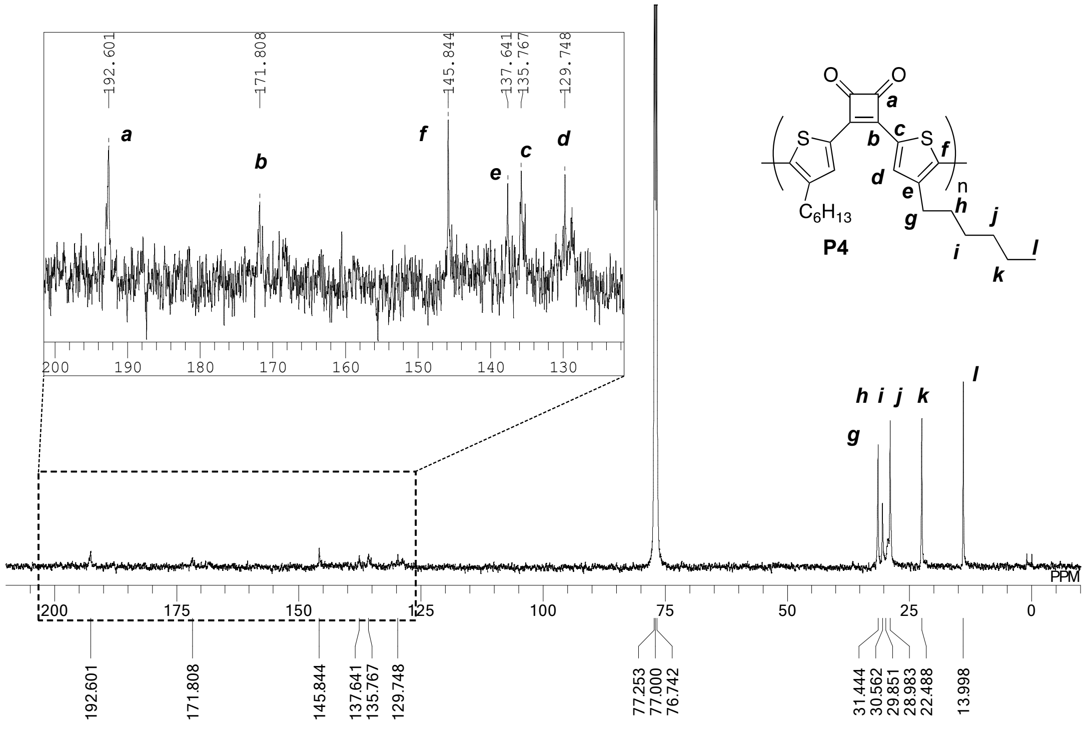

Figure S2. ^{13}C NMR spectra (126 MHz, $CDCl_3$) of **P4**.

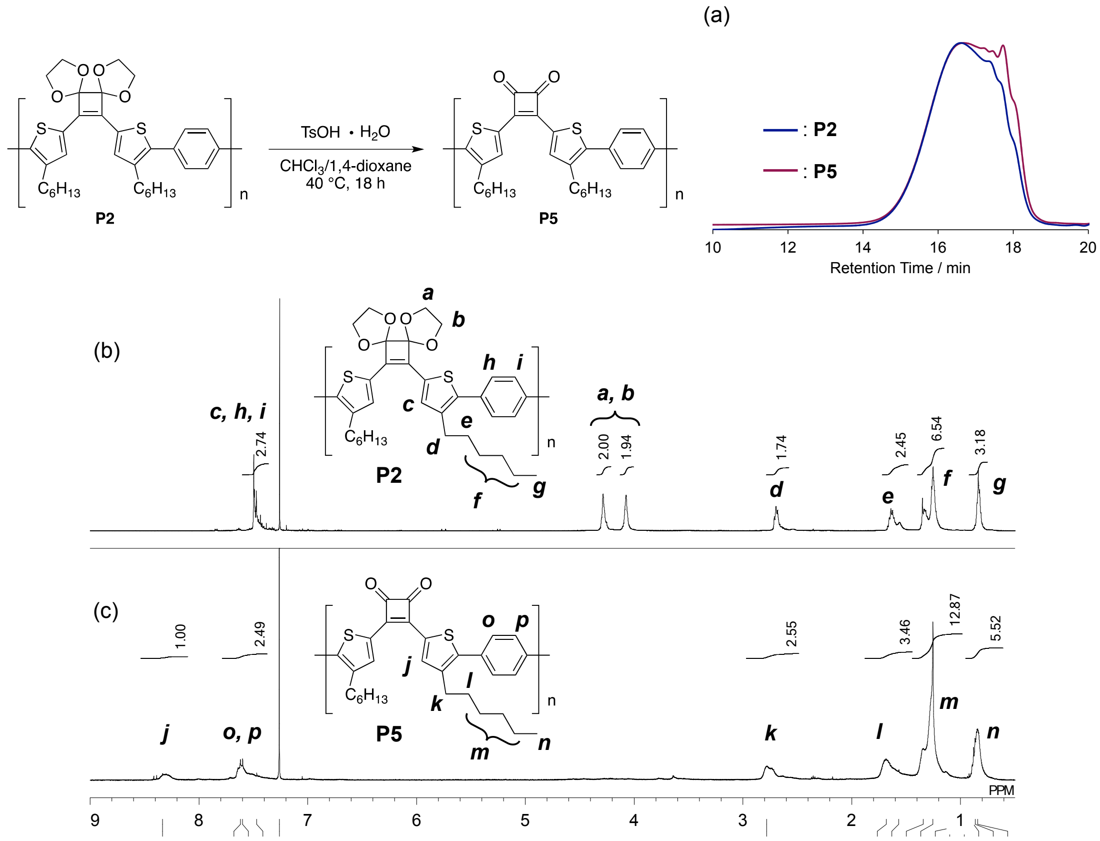

Figure S3. (a) GPC profiles of **P2** and **P5** (eluent: $CHCl_3$), and ^{1}H NMR spectra (500 MHz, $CDCl_3$) of (b) **P2** and (c) **P5**.

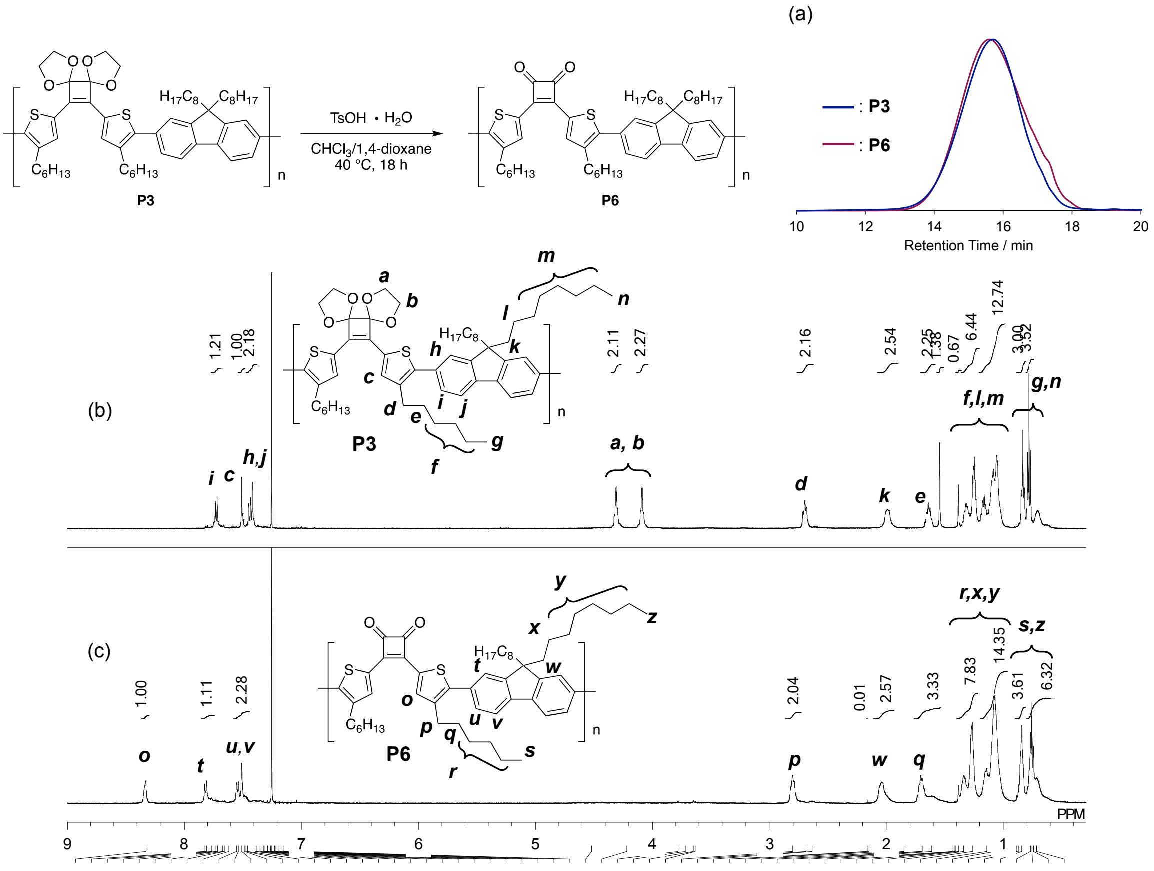

Figure S4. (a) GPC profiles of **P3** and **P6** (eluent: $CHCl_3$), and ^{1}H NMR spectra (500 MHz, $CDCl_3$) of (b) **P3** and (c) **P6**.

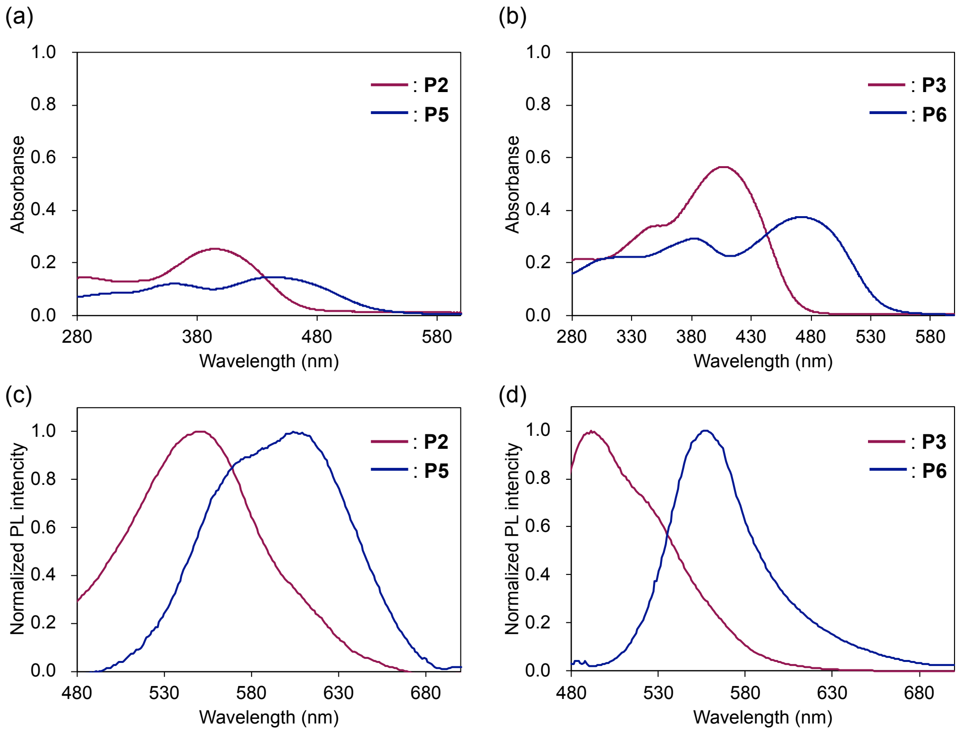

Figure S5. (a, b) UV-vis absorption and (c, d) photoluminescence spectra of **P2** (red), **P3** (red), **P5** (blue), and **P6** (blue) in $CHCl_3$ (1.0×10^{-5} M); PL excitation to the longest absorption maximum.

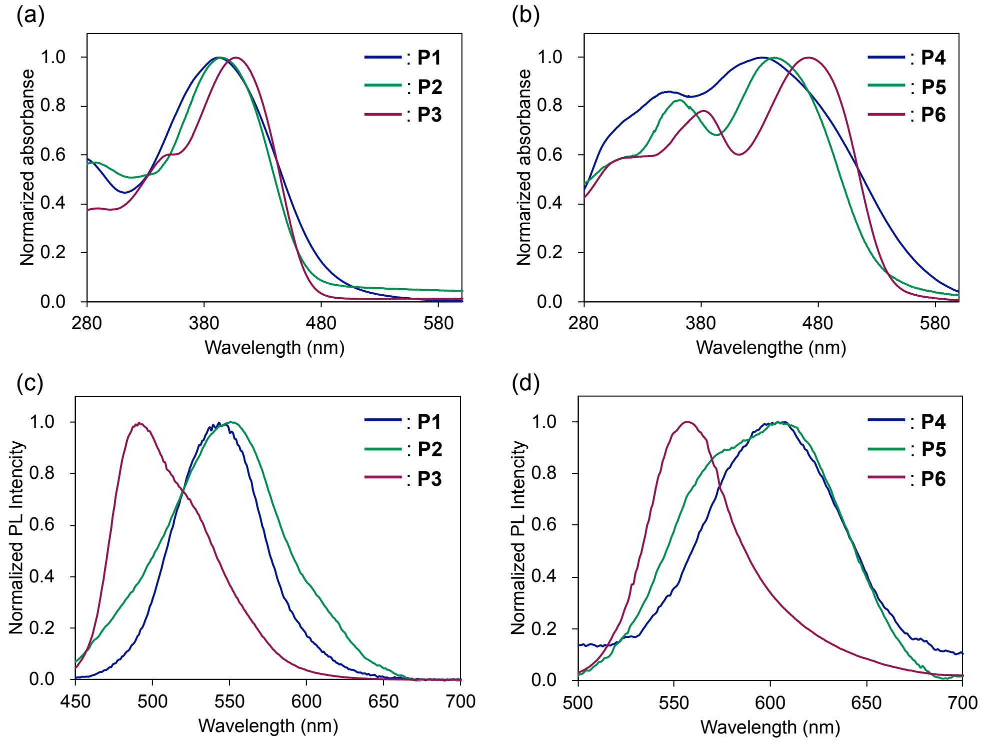

Figure S6. Normalized (a, b) UV-vis absorption and (c, d) photoluminescence spectra of **P1** (blue), **P4** (blue), **P2** (green), **P5** (green), **P3** (red), and **P6** (red) in $CHCl_3$ (1.0×10^{-5} M); PL excitation to the longest absorption maximum.

^{1}H NMR of **3b** (500 MHz, $CDCl_3$)

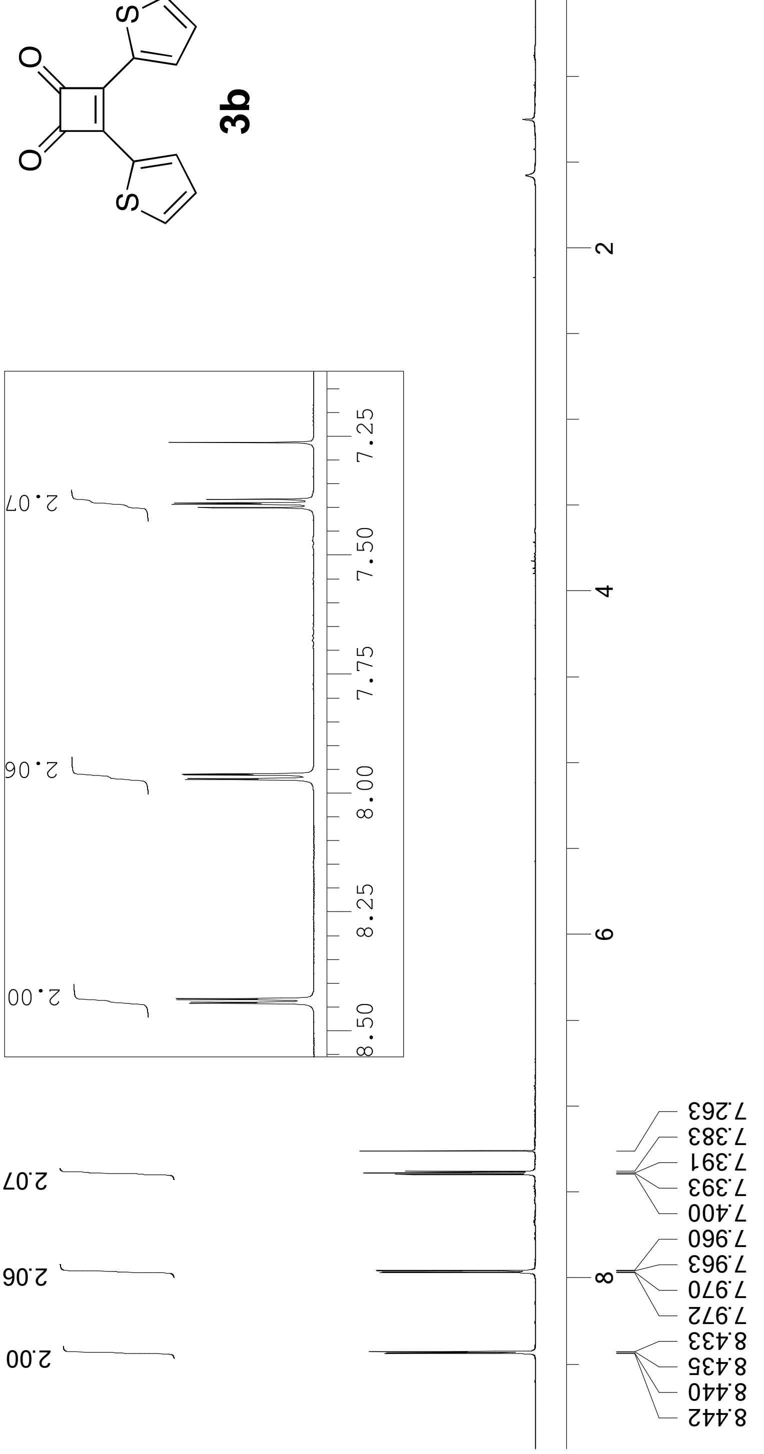

^{13}C NMR of **3b** (126 MHz, $CDCl_3$)

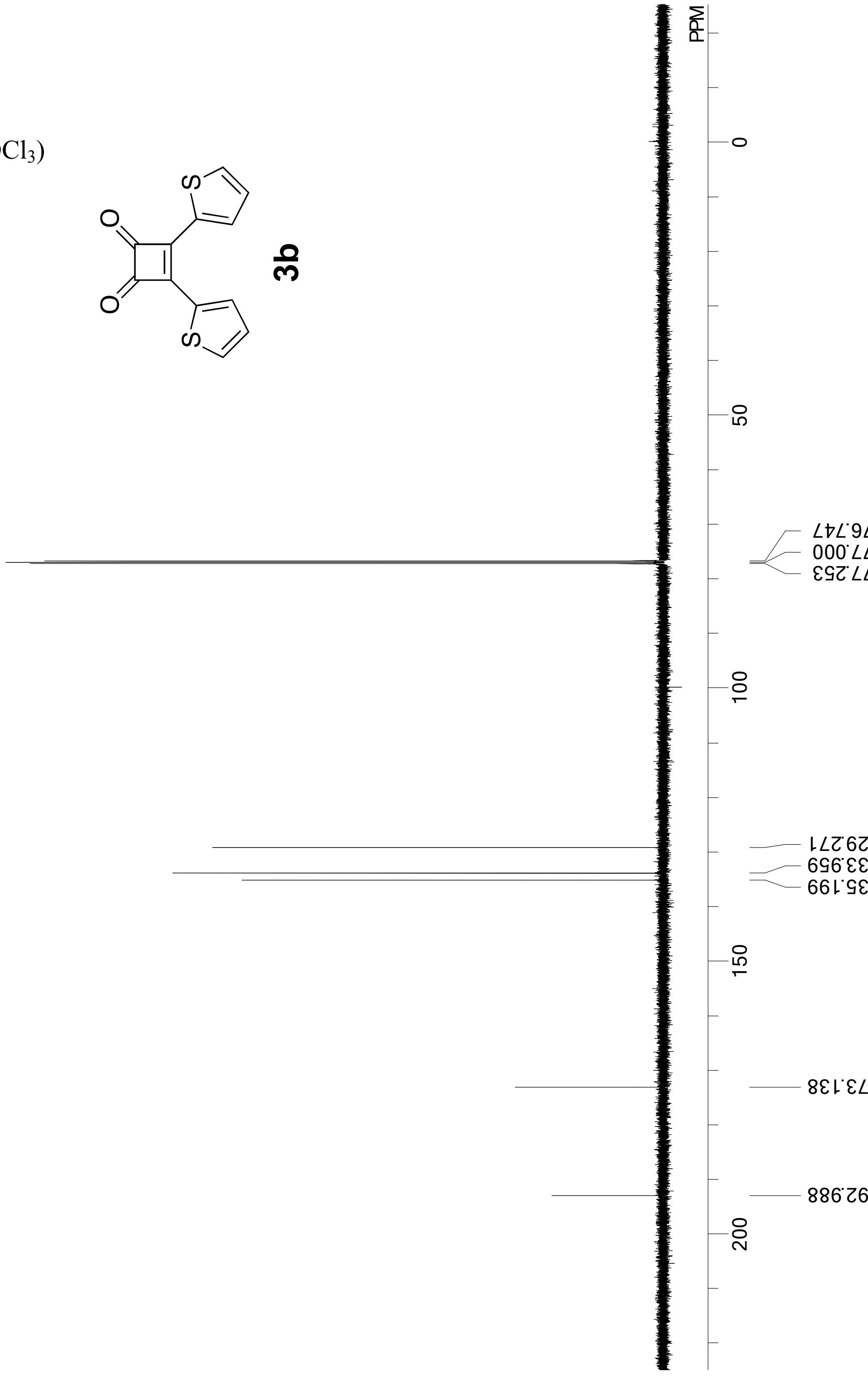

3b

^{1}H NMR of **4** (500 MHz, $CDCl_3$)

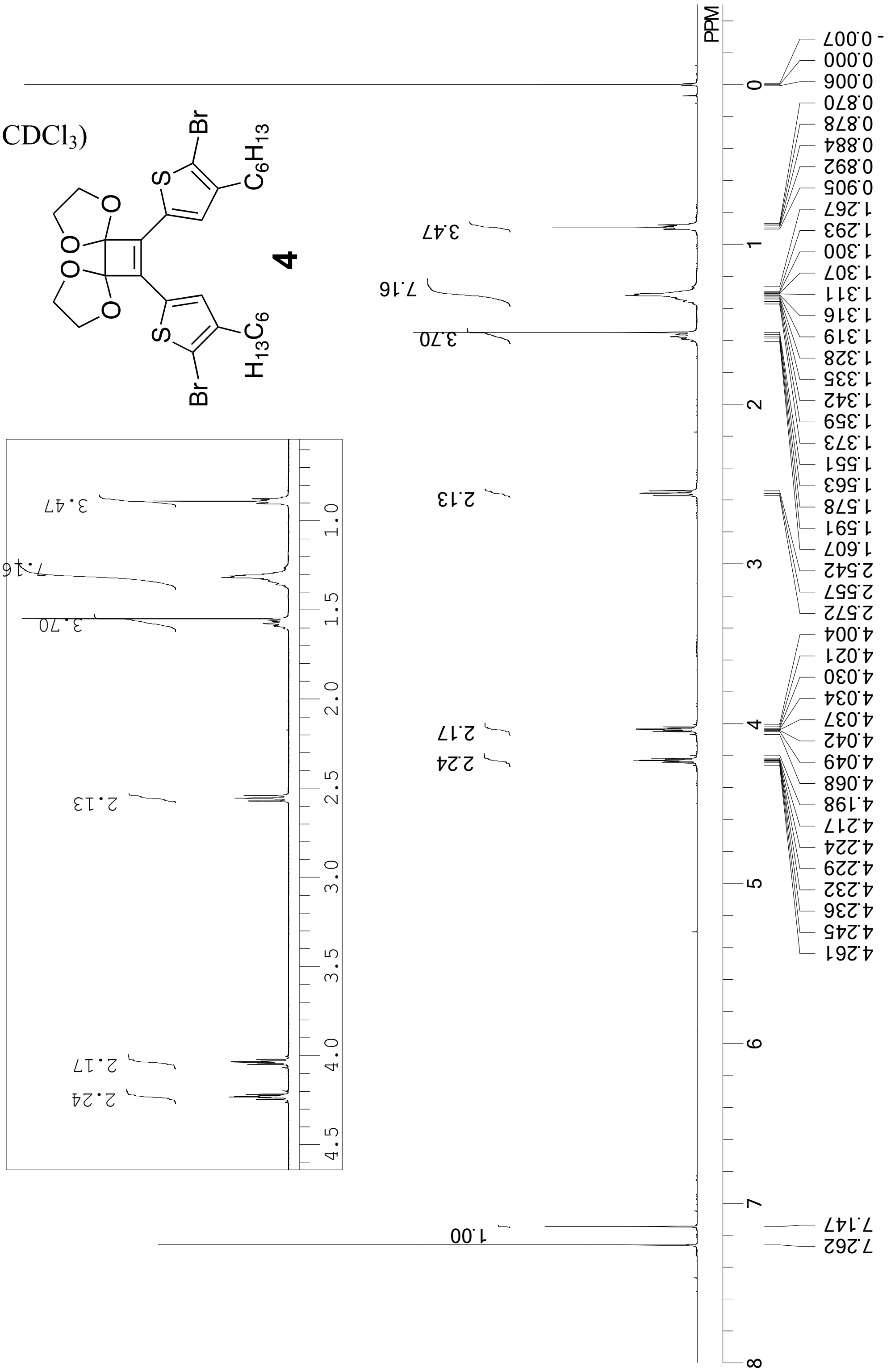

^{13}C NMR of **4** (126 MHz, $CDCl_3$)

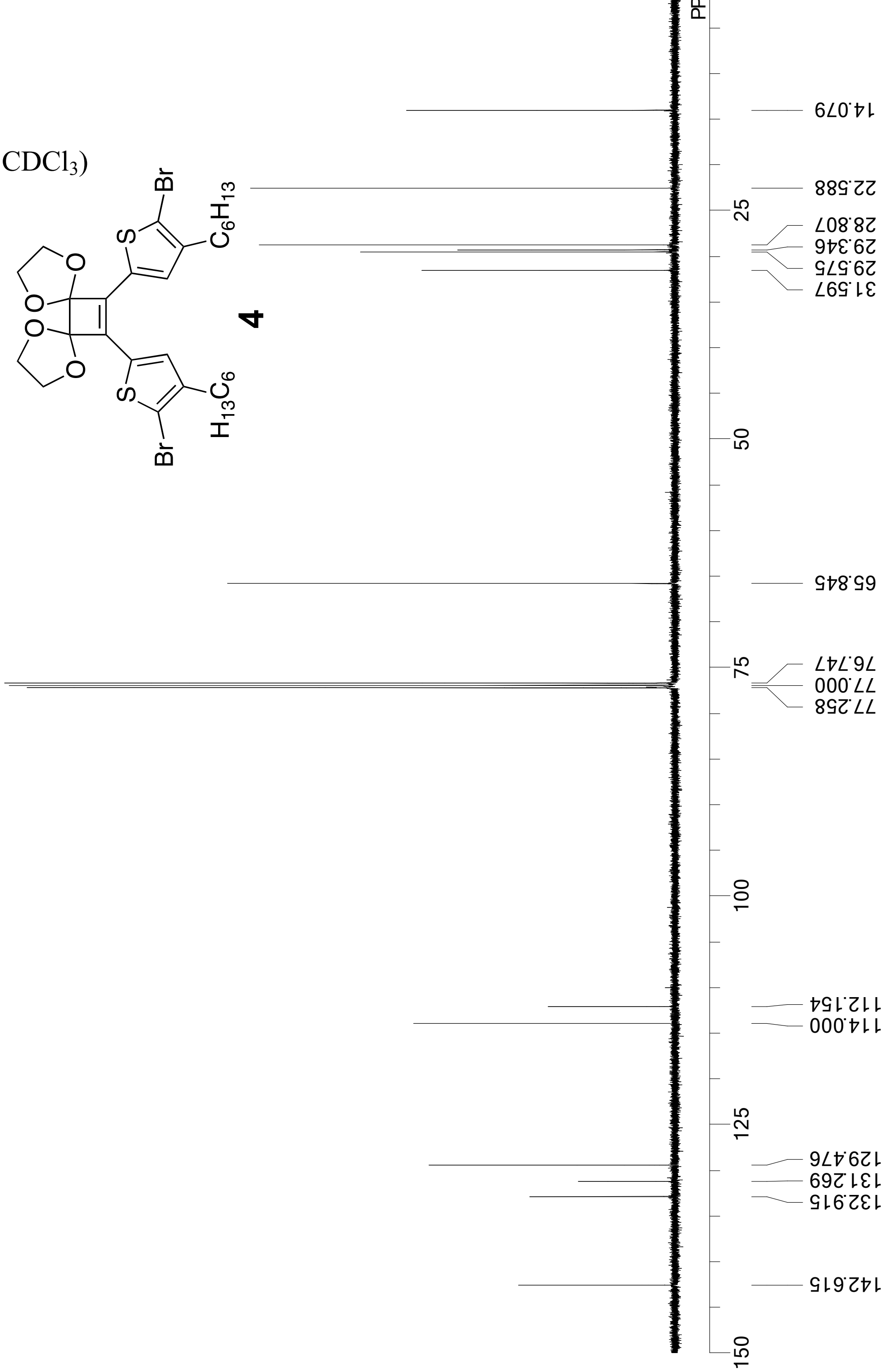

^{1}H NMR of **6** (500 MHz, $CDCl_3$)

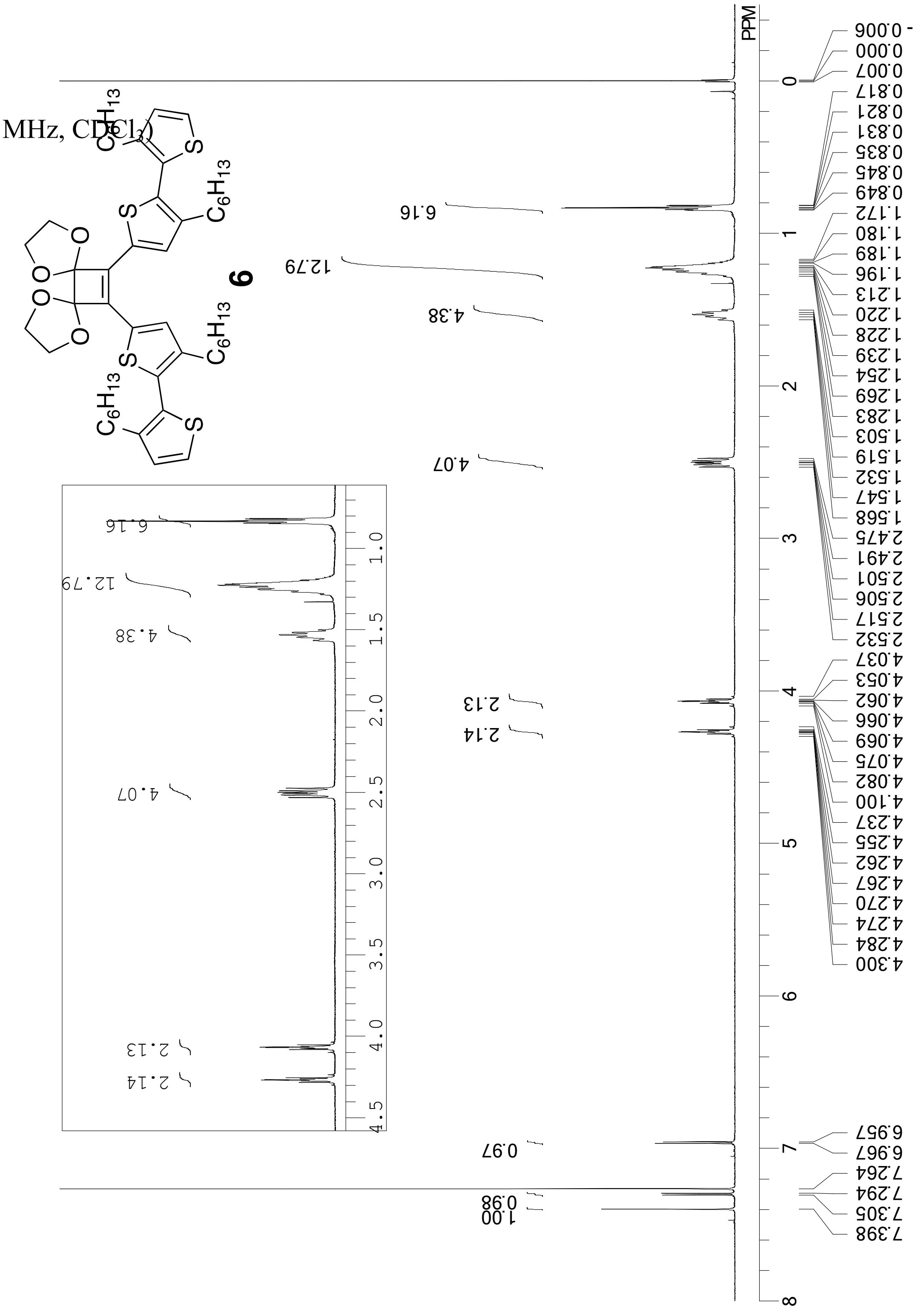

^{13}C NMR of **6** (126 MHz, $CDCl_3$)

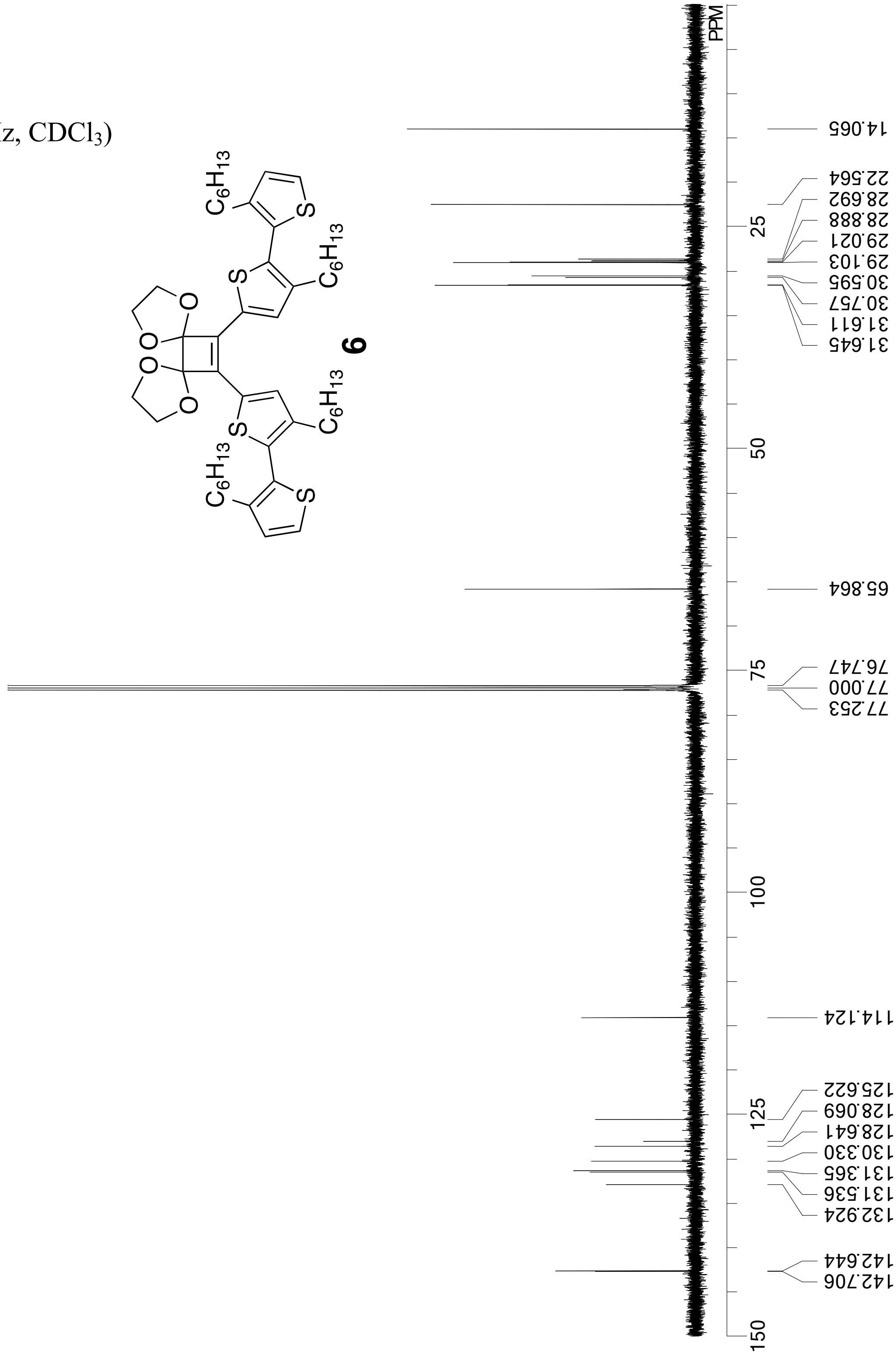

^{1}H NMR of **7** (500 MHz, $CDCl_3$)

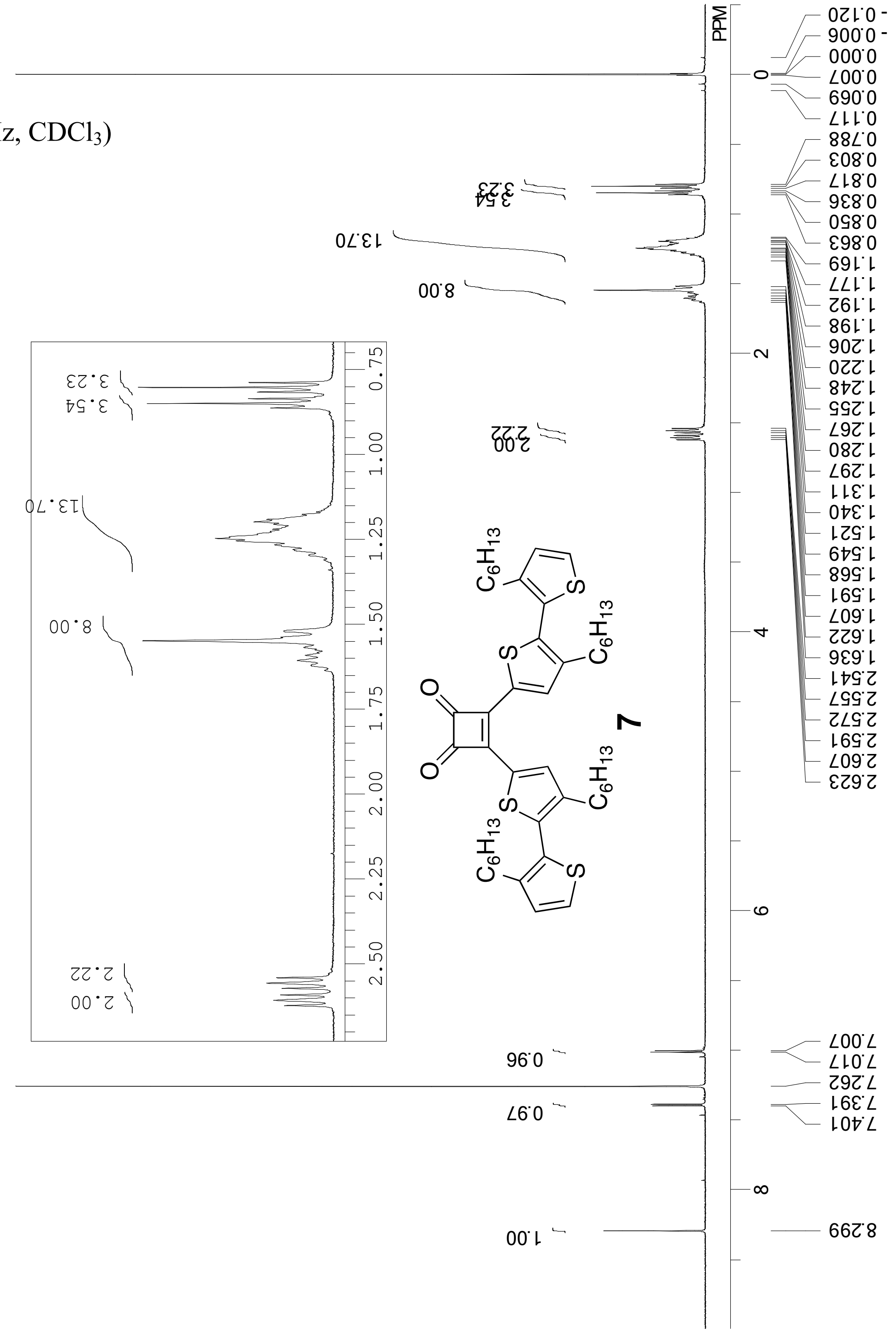

^{13}C NMR of **7** (126 MHz, $CDCl_3$)

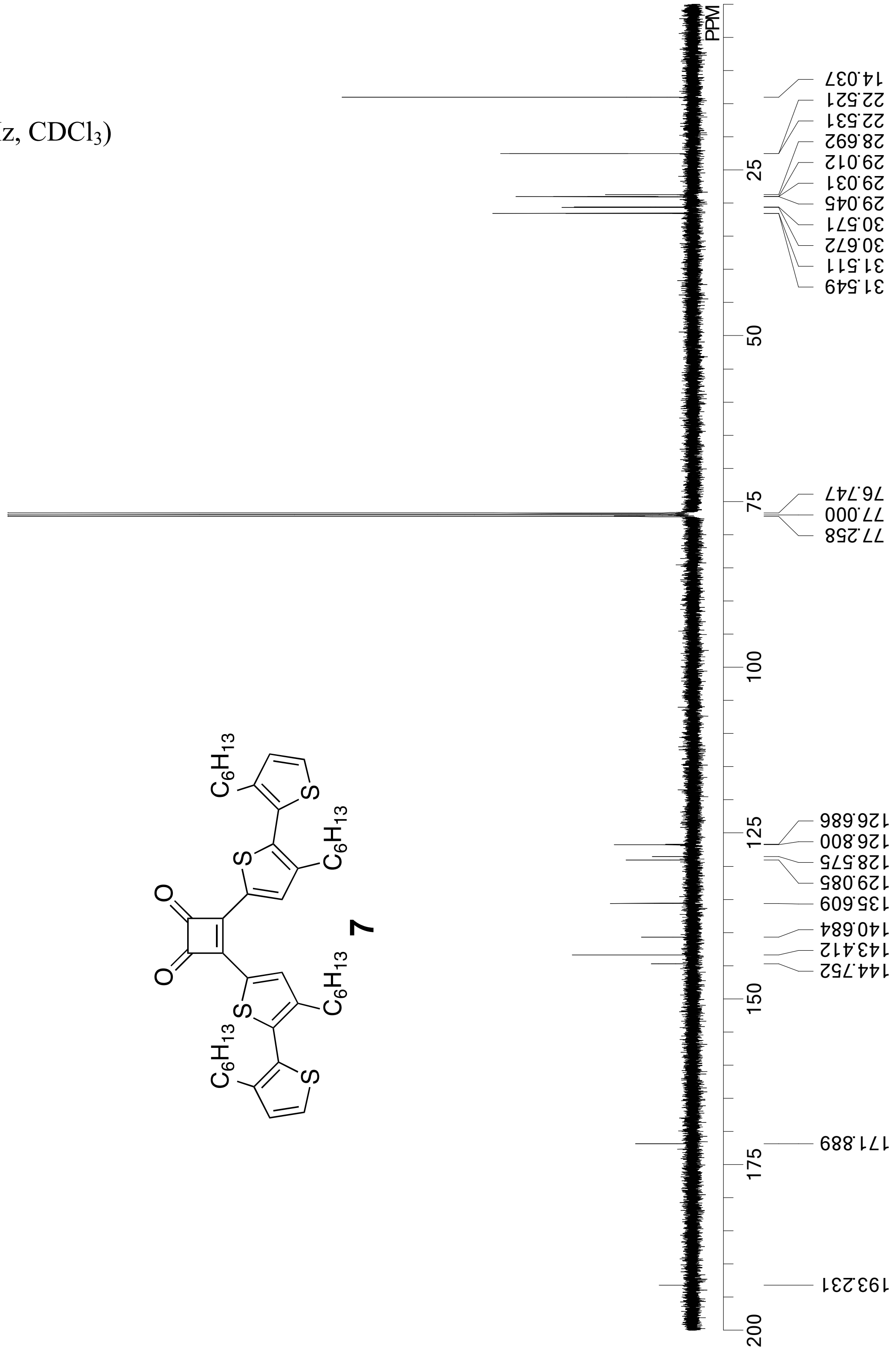

^{1}H NMR of **8** (500 MHz, $CDCl_3$)

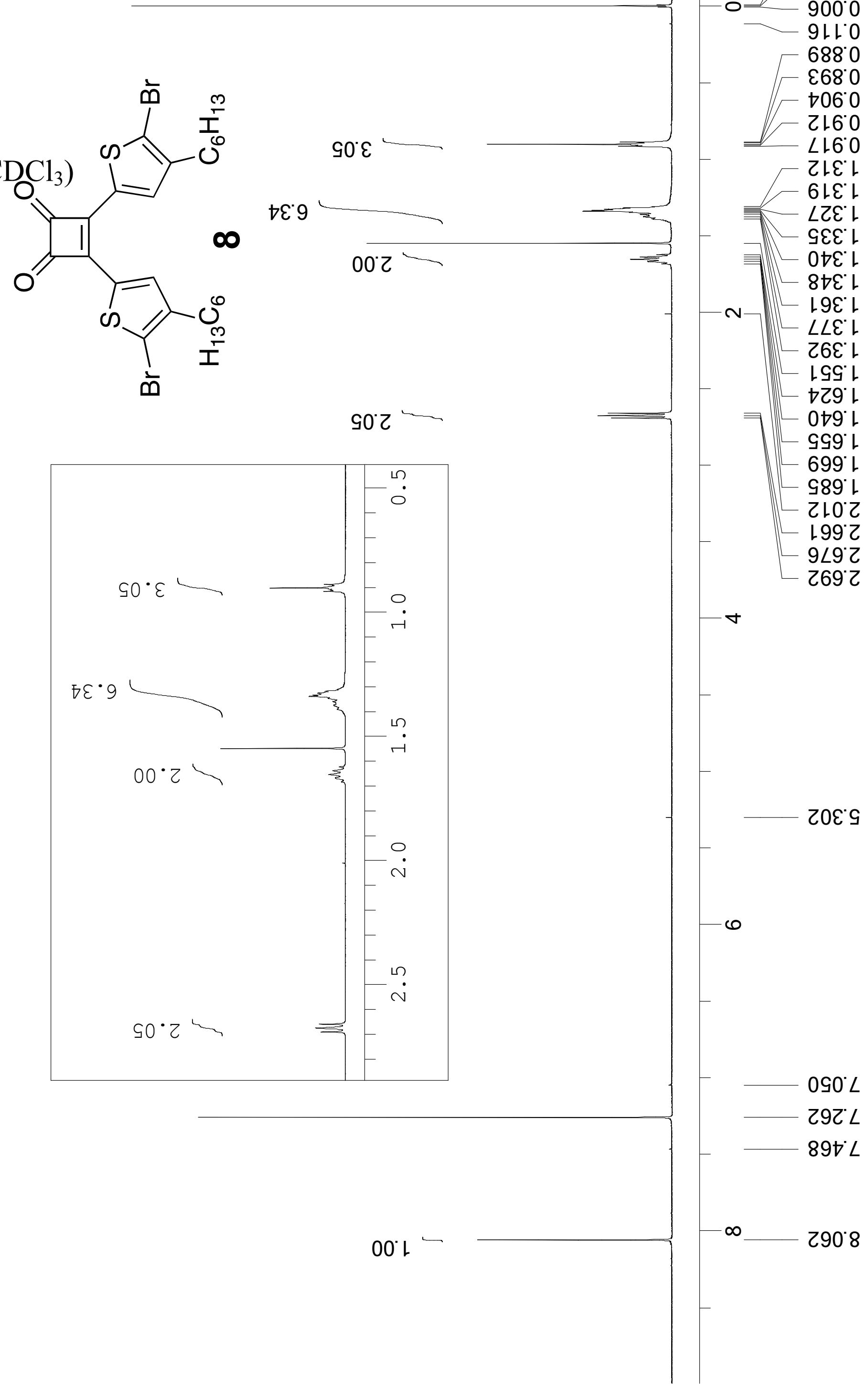

Supplement: RA-009-C9RA08275A-s001 [file RA-009-C9RA08275A-s001.pdf]
